# The protein kinase LKB1 negatively regulates bone morphogenetic protein receptor signaling

**DOI:** 10.18632/oncotarget.6683

**Published:** 2015-12-19

**Authors:** Erna Raja, Kalliopi Tzavlaki, Robin Vuilleumier, Karolina Edlund, Kaoru Kahata, Agata Zieba, Anita Morén, Yukihide Watanabe, Iryna Voytyuk, Johan Botling, Ola Söderberg, Patrick Micke, George Pyrowolakis, Carl-Henrik Heldin, Aristidis Moustakas

**Affiliations:** ^1^ Ludwig Institute for Cancer Research, Science for Life Laboratory, Uppsala University, Uppsala, Sweden; ^2^ Department of Medical Biochemistry and Microbiology, Science for Life Laboratory, Uppsala University, Uppsala, Sweden; ^3^ BIOSS, Centre for Biological Signaling Studies and Institute for Biology I, Faculty of Biology, Albert-Ludwigs-University of Freiburg, Freiburg, Germany; ^4^ Department of Immunology, Genetics and Pathology, Science for Life Laboratory, Uppsala University, Uppsala, Sweden

**Keywords:** BMP, differentiation, Drosophila, LKB1, lung cancer, Pathology Section

## Abstract

The protein kinase LKB1 regulates cell metabolism and growth and is implicated in intestinal and lung cancer. Bone morphogenetic protein (BMP) signaling regulates cell differentiation during development and tissue homeostasis. We demonstrate that LKB1 physically interacts with BMP type I receptors and requires Smad7 to promote downregulation of the receptor. Accordingly, LKB1 suppresses BMP-induced osteoblast differentiation and affects BMP signaling in *Drosophila* wing longitudinal vein morphogenesis. LKB1 protein expression and Smad1 phosphorylation analysis in a cohort of non-small cell lung cancer patients demonstrated a negative correlation predominantly in a subset enriched in adenocarcinomas. Lung cancer patient data analysis indicated strong correlation between LKB1 loss-of-function mutations and high BMP2 expression, and these two events further correlated with expression of a gene subset functionally linked to apoptosis and migration. This new mechanism of BMP receptor regulation by LKB1 has ramifications in physiological organogenesis and disease.

## INTRODUCTION

Bone morphogenetic proteins (BMPs) are evolutionarily conserved members of the transforming growth factor β (TGFβ) family that regulate cell growth, differentiation and apoptosis during development and adult tissue homeostasis [1-3]. The BMPs are implicated in diverse biological processes such as embryonic dorsoventral patterning and organogenesis, as for example in the *Drosophila melanogaster* wing and in the mammalian heart and kidney [2, 4, 5]. BMPs also induce differentiation of mesenchymal progenitor cells into mature osteoblasts or chondrocytes, thus contributing to the formation of bone and cartilage [4].

BMPs signal through binding to type I and type II transmembrane serine/threonine kinase receptors [6, 7]. Ligand binding allows the constitutively active type II receptor kinase to phosphorylate the type I receptor at its Gly/Ser-rich juxtamembrane domain, thus activating the kinase of the type I receptor. The BMP type II receptors consist of BMPRII, ActRIIA and ActRIIB, and the BMP type I receptors are BMPRIA (or activin receptor-like kinase 3; ALK3), BMPRIB (ALK6), ACVR1 (ALK2) and ACVRL1 (ALK1) [1, 3]. ALK1 and ALK2 are structurally similar to each other whereas ALK3 is highly similar to ALK6. Distinct BMP ligands have different binding affinities for the type I receptors. For instance, BMP2 and BMP4 preferentially bind to ALK3 and ALK6 [8] while BMP6 and BMP7 bind stronger to ALK2 and weaker to ALK6 [9]. In *Drosophila* a conserved set of signaling pathways operate in a similar manner as in mammals. Homo- and heterodimers of the BMP family ligands dpp (decapentaplegic), scw (screw), and gbb (glass bottom boat) signal *via* combinations of the type II receptors punt and wit (wishfull thinking) and the type I receptors tkv (thickveins) and sax (saxophone) [10, 11].

Ligand-activated BMP type I receptors phosphorylate the carboxyl-terminal Ser-X-Ser motifs in Smad1, Smad5 and Smad8 (receptor-activated (R-) Smads), and the phosphorylated R-Smads form complexes with Smad4 (common-mediator (co-) Smad) [6, 7]. Smad complexes accumulate in the nucleus and regulate the transcription of target genes. In *Drosophila*, the Smad signaling engine involves the R-Smad mad (mothers against decapentaplegic) and the co-Smad medea [5]. In addition to Smad signaling, the BMP receptors activate other signaling effectors, such as TAK1 (TGFβ-activated kinase 1), p38 MAPK (mitogen activated protein kinase) and JNK (c-Jun N-terminal kinase) [7, 12].

A widely studied cell model of BMP signaling is the pluripotent mouse myoblastic cell line C2C12, which differentiates into osteoblasts in response to many different BMPs [13]. *Id1* is transcriptionally induced by BMP Smad signaling during osteoblast differentiation and encodes a negative regulator of bHLH transcription factors [14, 15]. *Alkaline phosphatase* is also induced by BMP-activated MAPK and Smad pathways during osteoblastic differentiation [16]. In flies, a gradient of secreted dpp specifies the fly wing *via* transcriptional regulation by the mad/medea complex [17]. During pupal wing development, dpp ligand is expressed along longitudinal vein primordia and acts together with the broadly expressed ligand gbb to maintain and refine vein cell fates [5, 18].

BMP signaling can be negatively regulated by inhibitory (I) Smads, like Smad6 and Smad7, which bind the type I receptors and inhibit phosphorylation of R-Smads, and block the interaction between R-Smads and Smad4 [6, 7]. In addition, by recruiting the Smurf (Smad regulatory ubiquitinylation factor) ubiquitin ligases to the BMP type I receptors, I-Smads promote ubiquitinylation and degradation of the receptor complex [19].

LKB1 is a serine/threonine kinase that forms ternary complexes with the pseudokinase STRADα and the adaptor protein MO25 to create a catalytically active kinase [20]. LKB1 phosphorylates and enhances the catalytic activities of members of the AMP-regulated kinase (AMPK) family [21]. By controlling signaling *via* different AMPK family members, LKB1 regulates protein synthesis, cell proliferation, survival and polarity. LKB1 is classified as a tumor suppressor because loss of function mutations in LKB1 give rise to the Peutz-Jeghers syndrome, which is associated with benign gastrointestinal hamartomas and an elevated risk of developing carcinomas, including lung adenocarcinomas [22]. In *Drosophila*, the best established functions of lkb1 relate to the control of epithelial polarity and the establishment of the anterior-posterior body axis during embryogenesis [23]. In addition, fly lkb1 limits the growth of organs by activating JNK signaling [24]. The polarity pathway involves protein kinases of the AMPK family, such as MARKs (microtubule affinity-regulating kinases) and the prototype AMPKs, which control the activity of the PAR polarity complex and the proper orientation of the mitotic apparatus during early embryonic cell division [25, 26]. For this reason lkb1 null flies die very early during embryogenesis [25, 26]. *Via* distinct AMPK family members such as sik3 (salt-inducible kinase 3) *Drosophila* lkb1 also regulates adipocyte function and lipid metabolism [27].

Previous work has demonstrated that LKB1 induces secretion of TGFβ from mesenchymal cells, which then acts on neighboring epithelial cells in the gastrointestinal tract and limits their proliferation [28]. Loss of LKB1 in mesenchymal cells also leads to decreased differentiation of myofibroblasts due to reduced TGFβ secretion [29]. LKB1 can also negatively regulate TGFβ and BMP signaling as LKB1 inhibits the transcriptional function of Smad4 [30]. On the other hand, no link between lkb1 and dpp/scw/gbb signaling functions have been made in *Drosophila*.

In this article we investigated in more detail the crosstalk between LKB1 and BMP signaling, and establish that LKB1 negatively regulates BMP receptor function. The mechanism involves BMP type I receptor degradation in cooperation with Smad7.

## RESULTS

### LKB1 inhibits BMP-dependent gene expression and differentiation

**Figure 1:**
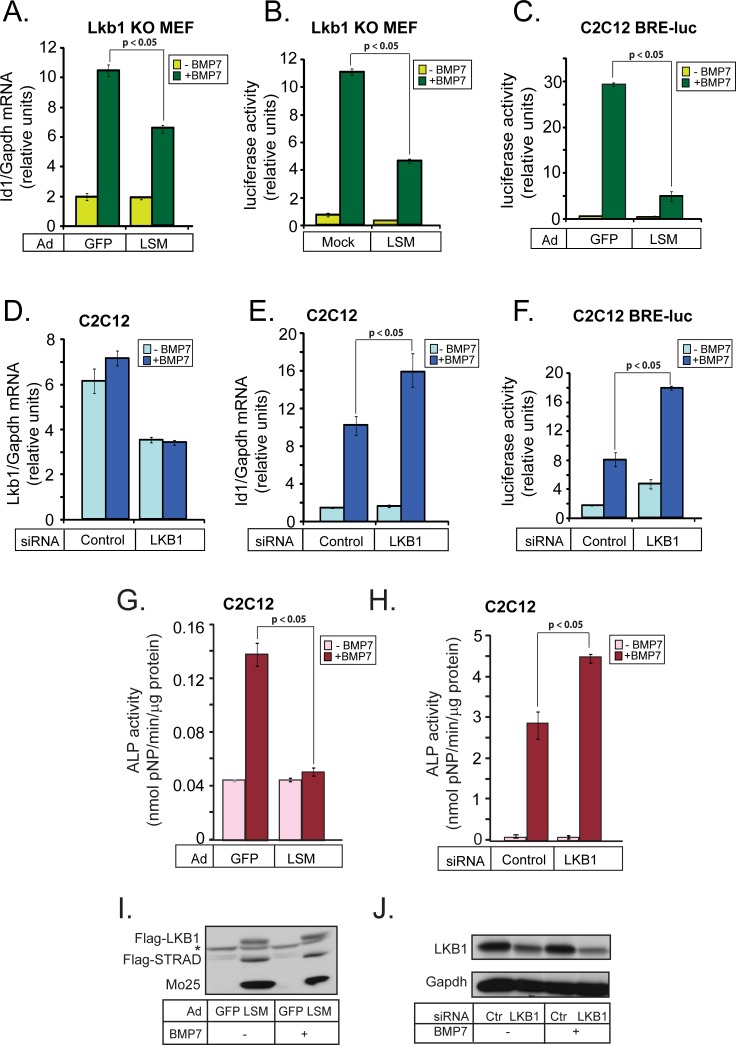
LKB1 inhibits BMP-induced gene expression and osteoblast differentiation **A.** Real-time RT-PCR analysis of endogenous *Id1* mRNA normalized to the corresponding *Gapdh* mRNA from *Lkb1* knockout (KO) MEFs infected with the indicated adenoviral vectors and stimulated with 30 ng/ml BMP7 for 19 h. Average values from triplicate determinations and the corresponding standard errors are graphed. **B.** Luciferase assay in *Lkb1* knockout (KO) MEFs infected with the indicated adenoviral vectors, transiently transfected with BRE_2_-luc reporter and stimulated with 30 ng/ml BMP7 for 24 h. The luciferase activity was normalized to the corresponding β-galactosidase activity. The bar graph shows average values derived from triplicate determinations and their corresponding standard errors. **C.** Luciferase assay in C2C12 cells stably expressing BRE_2_-luc reporter, performed as in panel B. **D.**, **E.** Real-time RT-PCR analysis of endogenous *Lkb1* and *Id1* mRNAs normalized to the corresponding *Gapdh* mRNA from mouse C2C12 cells transfected with the indicated siRNAs and stimulated with 30 ng/ml BMP7 for 24 h, presented as in panel A. **F.** Luciferase assay of C2C12 cells performed exactly as in panel B except that the cells were transfected with the indicated siRNAs and stimulated with 7.5 ng/ml BMP7. **G.** Alkaline phosphatase (ALP) assay in cell extracts of C2C12 cells infected with the indicated adenoviral vectors and stimulated with 300 ng/ml BMP7 for 3 days. Average enzymatic activity normalized to total protein per extract is plotted from triplicate determination with standard errors. **H.** Alkaline phosphatase assay in cell extracts of C2C12 cells transfected with the indicated siRNAs and stimulated with 300 ng/ml BMP7 for 3 days. The assay was performed as in panel G. In all panels, the BMP7-inducible mRNA, luciferase activity or ALP activity after LKB1 overexpression or silencing is shown in darker color. **I.** Immunoblot of the three adenovirally expressed proteins of the LSM complex in pooled C2C12 extracts analyzed in panel G. A star indicates a non-specific protein band. **J.** Immunoblot of endogenous LKB1 and Gapdh loading control protein in pooled C2C12 extracts analyzed in panel H. Statistical significance at *p* < 0.05 between the marked measurements is indicated.

Reproducing our previous work [30], we first verified that LKB1 robustly inhibits BMP signaling as represented by gene expression and cell differentiation. Thus, LKB1 negatively regulated expression of the mouse *Id1* gene and inducibility of an *Id1*-derived promoter-luciferase reporter (BRE_2_-luc) in response to BMP7 (Figure 1A-1F). Reconstitution of Lkb1 into mouse embryonic fibroblasts (MEFs) from *Lkb1* knockout mice together with its obligatory cofactors Stradα and Mo25 (LSM; Lkb1/Stradα/Mo25), reduced the physiological induction of endogenous *Id1* mRNA by BMP7 almost by half (Figure 1A), and also reduced the BMP7-induced activity of the BRE_2_ promoter (Figure 1B). The LSM triple protein expression method was preferred as the effects of reconstitution by single LKB1 were reproducibly weaker (see control experiments below). In an independent cell model, infection of mouse C2C12 pluripotent cells with the LSM adenoviral vectors dramatically suppressed BRE_2_ promoter activity (Figure 1C). To test the importance of endogenous mouse Lkb1 in the same signaling processes, we silenced endogenous Lkb1 by 50% using short interfering RNA (siRNA) transfection in C2C12 cells (Figure 1D), and found a 1.7- to 2-fold increase in the levels of endogenous *Id1* mRNA after BMP7 stimulation (Figure 1E). Endogenous Lkb1 silencing also led to a doubling of the responsiveness of the BRE_2_ promoter (Figure 1F).

One of the hallmark responses of mesenchymal progenitor cells, such as C2C12 cells, to BMP signaling is their differentiation to osteoblasts, which is classically measured as accumulation of alkaline phosphatase (Alp) activity [13] (Figure 1G). Transduction of C2C12 cells with LSM viruses (Figure 1I), almost completely abolished differentiation as Alp activity measured in the presence of BMP7 was low, similar to control cells (Figure 1G). On the other hand, silencing of endogenous Lkb1 further potentiated differentiation (Figure 1H; the efficiency of mouse Lkb1 protein silencing is shown in Figure 1J). We conclude that Lkb1 suppresses BMP signaling with an impact on physiological gene responses to BMP, and limits the capacity of BMP to induce osteoblastic differentiation in mesenchymal progenitor cells.

### LKB1 negatively regulates BMP R-Smad C-terminal phosphorylation

**Figure 2:**
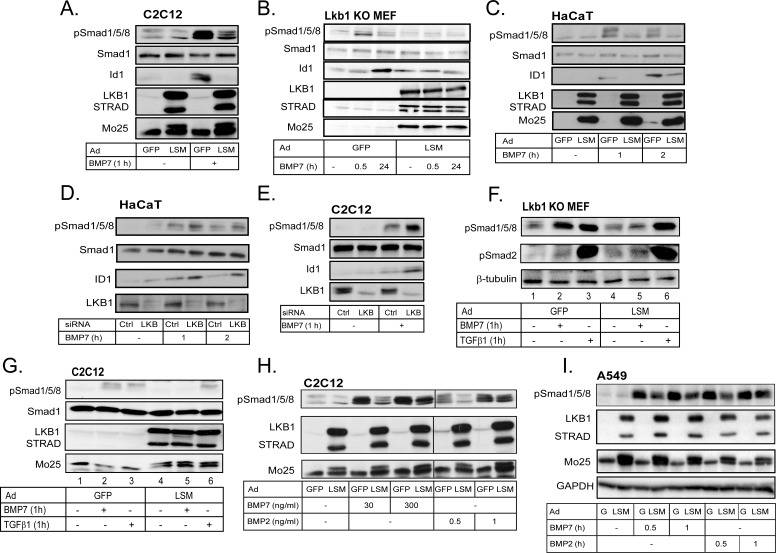
LKB1 suppresses BMP-induced phosphorylation of Smad1/5/8 **A.**-**C.** Immunoblot of endogenous phospho-Smad1/5/8, Smad1 and Id1 and of the three adenovirally expressed proteins of the LSM complex in extracts of C2C12 (A), *Lkb1* KO MEF (B) and HaCaT (C) cells after stimulation with BMP7 (30 ng/ml) for the indicated time periods. **D.**, **E.** Immunoblot of endogenous phospho-Smad1/5/8, Smad1, ID1 and LKB1 in extracts of HaCaT (D) and C2C12 (E) cells after transfection of the indicated siRNAs and stimulation with BMP7 (30 ng/ml) for the indicated time periods. **F.** Immunoblot of endogenous phospho-Smad1/5/8, phospho-Smad2 and β-tubulin proteins in *Lkb1* knockout MEF extracts after adenoviral infection and stimulation with BMP7 (30 ng/ml) or TGFβ1 (5 ng/ml) for 1 h. **G.** Immunoblot of endogenous phospho-Smad1/5/8 and Smad1 proteins and of the three adenovirally expressed proteins of the LSM complex in C2C12 extracts after stimulation with BMP7 (30 ng/ml) or TGFβ1 (5 ng/ml) for 1 h. **H.** Immunoblot of endogenous phospho-Smad1/5/8 and adenovirally expressed LSM proteins in extracts of C2C12 cells after infection and stimulation with the indicated concentrations of BMP2 and BMP7 for 1 h. **I.** Immunoblot of endogenous phospho-Smad1/5/8, GAPDH (loading control) and adenovirally expressed LSM proteins in extracts of A549 cells after infection and stimulation with BMP7 (30 ng/ml) or BMP2 (10 ng/ml) for the indicated time periods.

In addition to inhibition of Smad4 binding to DNA [30], LKB1 could affect early BMP signaling at the level of R-Smad phosphorylation by type I receptors. We found that LKB1 strongly suppressed BMP7-dependent R-Smad phosphorylation (Figure 2). In pluripotent C2C12 cells BMP7 stimulation induced robust C-terminal phosphorylation of Smad1, Smad5 and Smad8, and LSM expression downregulated the signal (Figure 2A). Activation of phospho-Smads was quickly followed by accumulation of the Id1 protein after *Id1* gene induction, and LSM strongly blocked Id1 protein levels in the C2C12 cells (Figure 2A). In the *Lkb1* knockout MEFs infected with control GFP virus, phospho-Smad1/5/8 was induced by 30 min of BMP7 stimulation and decreased after 24 h, and endogenous Id1 protein accumulated at 30 min followed by higher levels at 24 h (Figure 2B). When the knockout cells were transduced with LSM, the BMP7-inducible phospho-Smad1/5/8 or Id1 levels were completely suppressed at both time points (Figure 2B).

In order to validate the findings from the two independent mouse cell models to a human system, we employed human HaCaT keratinocytes that are very well established for their signaling and physiological responses to all members of the TGFβ family including the BMPs [31], and human lung adenocarcinoma A549 cells, which also respond well to TGFβ family members and provide relevance to lung cancer (see below) [32]. Similar to the above results were measured in HaCaT keratinocytes, but for different times (1 and 2 h) of stimulation with BMP7, (Figure 2C). In all three cell models the levels of total Smad1 did not appreciably change in response to BMP7 signaling or in response to LSM expression (Figure 2A-2C). Using the *Lkb1* knockout MEFs, the HaCaT and A549 cells we also demonstrated that expression of single LKB1 kinase components, LKB1, STRADα or MO25 could lead to weaker but detectable downregulation of BMP7 signaling, however, the efficiency was dramatically enhanced when all three kinase components were co-expressed in the form of LSM (Supplementary Figure 1A-1C). After silencing of endogenous mouse Lkb1 in C2C12 cells and human LKB1 in HaCaT cells using siRNA, we measured significantly enhanced phosphorylation of BMP R-Smads and enhanced BMP7-inducible levels of ID1, without measurable effects on total Smad1 levels (Figure 2D, 2E).

To investigate how LKB1 inhibits C-terminal phosphorylation of BMP R-Smads, we tested if Lkb1 could affect the stability of phospho-Smad1/5/8 in *Lkb1* knockout MEFs (Supplementary Figure 1D). The proteasomal inhibitor MG132 and the lysosomal inhibitor chloroquine, stabilized the levels of phospho-Smad1/5/8 after stimulation with BMP7 and also stabilized the basal phospho-Smad1/5/8 levels even in the absence of stimulation (Supplementary Figure 1D, lanes 5, 6). However, chloroquine and MG132 did not rescue the effect of LSM on phospho-Smad1/5/8 suppression (Supplementary Figure 1D, lanes 7, 8), which suggests that Lkb1 does not promote directly the degradation of phospho-Smad1/5/8. Next, we examined the possibility that Lkb1 caused dephosphorylation of R-Smads or possibly of the type I receptors *via* a mechanism involving phosphatases; we found that okadaic acid, an inhibitor of serine/threonine phosphatases [33], enhanced and prolonged the phosphorylation of Smad1/5/8 after BMP7 stimulation (Supplementary Figure 1E, lanes 3, 9). However, LSM still potently inhibited BMP7-induced phospho-Smad1/5/8 in the presence of okadaic acid, suggesting a phosphatase-independent mechanism (Supplementary Figure 1E, lanes 4-6, 10-12). LKB1 is known to act as a central mediator of AMPK signaling but can also phosphorylate and regulate substrates directly in the absence of AMPKs [21]. We therefore stimulated C2C12 cells with AICAR, an established AMP analog that activates the endogenous AMPKs (Supplementary Figure 1F). AICAR activated endogenous phosphorylated and active form of mouse Ampk, however, it could not significantly downregulate BMP signaling as analyzed by phospho-Smad1/5/8 immunoblot (Supplementary Figure 1F). The LKB1-AMPK pathway is also known to lead to activation of the mTOR kinase that controls cell metabolism and protein synthesis [21]. Using rapamycin, a general mTOR kinase inhibitor, we could effectively block endogenous mTor kinase signaling, measured by complete block of phosphorylated p70 S6 kinase levels (Supplementary Figure 1G). However, rapamycin did not block BMP7-induced phospho-Smad1/5/8 levels (Supplementary Figure 1G). Finally, since we have previously shown that TGFβ receptor signaling is negatively regulated by the AMPK family kinase SIK (salt-inducible kinase) [34], we also tested whether SIK could replace LKB1 and block BMP signaling. Expression of the active LSM complex in C2C12 cells fully blocked BMP7-induced phospho-Smad1/5/8, whereas expression of SIK under the same conditions had no impact on BMP7 signaling (Supplementary Figure 1H). All these experiments suggested that LKB1 functions in the absence of assistance from downstream AMPKs, and that LKB1 acts downstream of BMP7 and upstream of R-Smad phosphorylation, pointing to the BMP receptors.

### LKB1 acts at the level of BMP type I receptors

In order to elucidate whether LKB1 affects the BMP receptors, we took advantage of the fact that, in addition to BMPs, TGFβ signaling can also induce phosphorylation of Smad1/5/8 under certain conditions [35-37]. We therefore compared side-by-side stimulation of *Lkb1* knockout MEFs and C2C12 cells with BMP7 and TGFβ1 for 1 h (Figure 2F, 2G). Interestingly, transduction of the cells with LSM led to a complete inhibition of BMP7-induced phosphorylation of Smad1/5/8 (Figure 2F, 2G), but had no impact on the TGFβ1-induced phosphorylation of Smad1/5/8 (Figure 2F, 2G). In the same experiment (Figure 2F), we also verified that Lkb1 failed to repress the phosphorylation of Smad2 in response to TGFβ1. These experiments suggest that LKB1 specifically suppresses the accumulation of phosphorylated R-Smads in response to BMP and fails to affect the phosphorylation of R-Smads in response to TGFβ; therefore, LKB1 may regulate BMP receptor activity or stability.

Since Smad1/5/8 C-terminal phosphorylation is catalyzed by the BMP type I receptors, we then attempted to get a first look at the BMP type I receptor whose signaling might be targeted by LKB1. To achieve this we used the mouse C2C12 cells that express only Acvr1/Alk2 and Bmpr1A/Alk3, but not Bmpr1B/Alk6 [16]. We compared two distinct ligands of the BMP family; BMP2, which signals mainly *via* Bmpr1A/Alk3 and Bmpr1B/Alk6 [8], and BMP7, signaling mainly *via* Acvr1/Alk2 and less *via* Bmpr1B/Alk6 [9, 38]. Transduction of C2C12 cells with LSM and stimulation with increasing concentration of each ligand demonstrated that Lkb1 inhibited phosphorylation of Smad1/5/8 induced by either ligand (Figure 2H). Lkb1 was competent to partially suppress phospho-Smad1/5/8 induced by a 10-fold higher dose of BMP7 (300 ng/ml) compared to the standard dose used in most experiments (30 ng/ml). In contrast, increasing the BMP2 dose just by 2-fold (from 0.5 to 1 ng/ml) exhibited robust phosphorylation levels of Smad1/5/8 despite the presence of the inhibitory Lkb1 (Figure 2H). The doses of BMP2 were different from those of BMP7 as preliminary titration experiments had revealed that 10-30 ng/ml BMP7 gave a similar level of phospho-Smad1/5/8 as 1-2 ng/ml BMP2 in this cell model. This experiment suggested that Lkb1 may regulate more efficiently AcvR1/Alk2 compared to Bmpr1A/Alk3 in the mouse C2C12 cells as these cells do not express the Bmpr1B/Alk6 receptor. Similar results were obtained in the human lung adenocarcinoma A549 cell model, in which LKB1 inhibited BMP7-induced phospho-Smad1/5/8 more potently and in a more sustained manner, compared to the effect LKB1 had against BMP2 signaling; this effect was stronger at very early time points and weaker at later time points (Figure 2I). Thus, LKB1 might also block human ACVRI/ALK2 signaling more efficiently than BMPRIA/B signaling.

**Figure 3:**
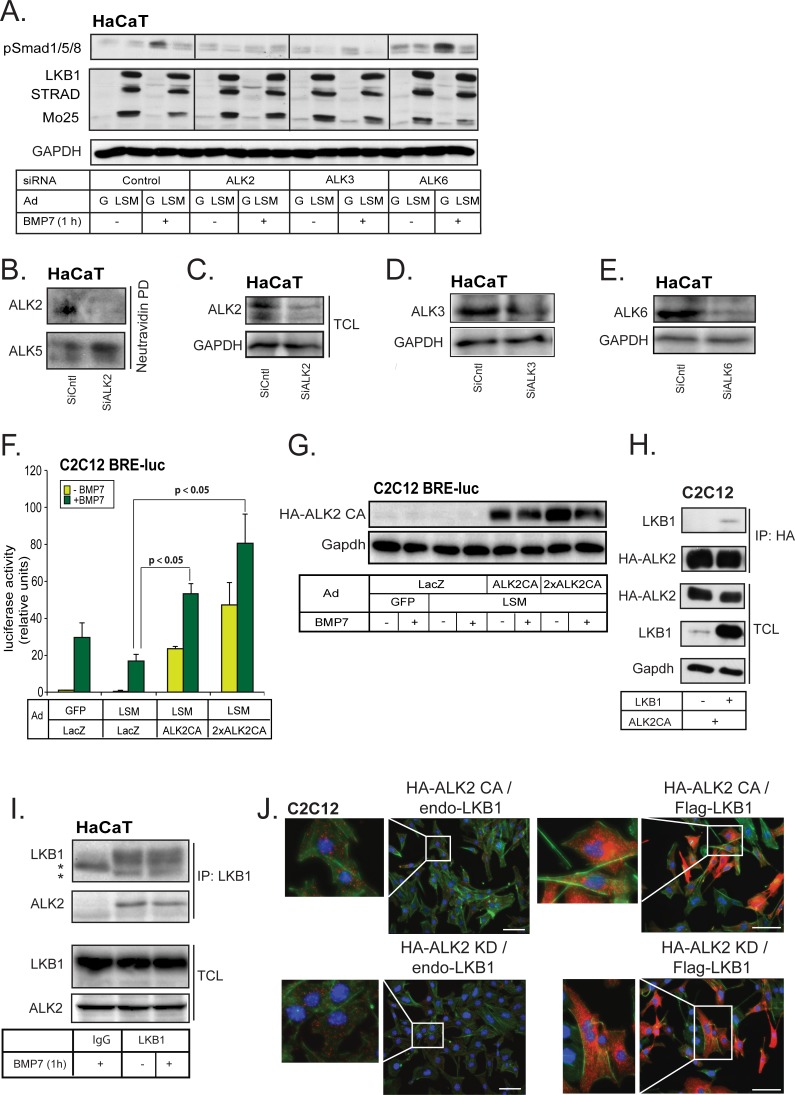
LKB1 crosstalks and interacts with BMP type I receptor **A.** Immunoblots of endogenous phospho-Smad1/5/8, GAPDH (loading control) and adenovirally expressed LSM proteins in HaCaT extracts after transfection with the indicated receptor siRNAs and stimulation with 30 ng/ml BMP7 for 1 h. **B.**, **C.** Immunoblots measuring total (C) and cell surface expression (B) of endogenous ALK2 in HaCaT cells after transfection with the ALK2 receptor siRNA. After cell surface biotinylation, the surface proteins were pulled-down (PD) with neutravidin prior to immunoblot analysis. GAPDH serves as a loading control for total cell lysates (C) and ALK5 as control for biotinylated cell surface fractions (B) and lack of off-target effects of the siRNA. **D.**, **E.** Immunoblots measuring total expression of endogenous ALK3 (D) and ALK6 (E) in HaCaT cells after transfection with the respective receptor siRNA. GAPDH serves as a loading control. **F.** Luciferase assay in C2C12 cells stably expressing BRE_2_-luc reporter and stimulated with 30 ng/ml BMP7 for 24 h after infection with the indicated adenoviral vectors. The luciferase activity was normalized to the corresponding β-galactosidase activity. One specific dose and twice as much (2×) of the ALK2 CA virus were used for infection. Statistical significance at *p* < 0.05 between the marked measurements is indicated. **G.** Immunoblot of adenovirally expressed constitutively active (CA) ALK2 receptor and Gapdh (loading control) in C2C12 BRE_2_-luc extracts analysed in panel F. **H.** Co-immunoprecipitation assay of HA-tagged ALK2 with LKB1 in transfected C2C12 cells. HA antibody was used to pull down ALK2, and Gapdh blot indicates equal protein amounts in the total cell lysates (TCL). **I.** Co-immunoprecipitation assay of endogenous LKB1 with endogenous wild-type ALK2 receptor in the absence (−) or presence (+) of BMP7 stimulation for 1 h in HaCaT cells. Extracts were immunoprecipitated with LKB1 antibody or control mouse IgG and immunoblotted for ALK2 and LKB1. Total cell lysates (TCL) were immunoblotted with the same antibodies. Stars indicate the respective heavy immunoglobulin protein bands used for immunoprecipitation. **J.** Immunocomplexes (red dots) detected using proximity ligation assay in C2C12 cells using anti-HA and anti-LKB1 antibodies. Green color represents the actin cytoskeleton stained with phalloidin to mark overall cell morphology and integrity, and blue color represents nuclear staining with Hoechst. Cells were transduced with constitutively active ALK2 (top left), kinase-dead ALK2 (bottom left), both constitutively active ALK2 and LKB1 (top right), or both kinase-dead ALK2 and LKB1 (bottom right). Bars indicate 10 μm and insets show higher magnification of selected details.

We then investigated further the human epithelial HaCaT cells that express relatively comparable levels of all three BMP type I receptors. Silencing of each type I receptor separately and stimulating the cells with BMP7 showed that in these cells, BMP7 signals *via* ACVR1/ALK2, as expected, but also *via* BMPR1A/ALK3, as knockdown of either of these receptors inhibited the phosphorylation of Smad1/5/8; in contrast silencing of BMR1B/ALK6 did not have an impact on the phospho-Smad1/5/8 level after BMP7 stimulation (Figure 3A). The efficiency of knockdown of each type I receptor was about 75% in each case at the mRNA level (Supplementary Figure 2A). A 75% or greater silencing efficiency of total endogenous ALK2 receptor was observed by immunoblotting. Moreover, a higher than 80% silencing efficiency of cell surface endogenous ALK2 receptor was observed, as determined by biotinylation of surface proteins, followed by neutravidin pull-down and immunoblotting, without evident off-target effects of the siRNA towards a distinct type I receptor, ALK5 (Figure 3B, 3C). Similarly, the silencing efficiency of endogenous BMPR1A/ALK3 at the protein level was more than 60% and for BMPR1B/ALK6 more than 75% in HaCaT cells (Figure 3D, 3E).

We then investigated the effect of silencing each of the endogenous BMP type I receptors in HaCaT cells infected simultaneously with LSM (Figure 3A). LSM suppressed the phosphorylation of Smad1/5/8 after control siRNA transfection, demonstrating that the cells responded to the LKB1 signals under the combined conditions of viral infection and siRNA transfection (Figure 3A). When ACVR1/ALK2 was silenced, BMP7 induced minimal phospho-Smad1/5/8 levels and LSM was ineffective in further reducing these low levels of phospho-Smad1/5/8. When BMPR1A/ALK3 was silenced, BMP7 induced low phospho-Smad1/5/8 level as well, but LSM effectively reduced this further to background level. Finally, when BMPR1B/ALK6 was silenced, BMP7 still induced a robust phospho-Smad1/5/8 level and LSM reduced it significantly (Figure 3A). The same experimental setting was repeated, but the HaCaT cells were stimulated with BMP2 (Supplementary Figure 2B). Silencing of ALK3 had a strong impact on Smad1/5/8 phosphorylation in this cell type (Supplementary Figure 2B), whereas silencing of ALK6 had a weaker effect and silencing of ALK2 had a measurable but even weaker impact compared to the ALK3 siRNA (Supplementary Figure 2B). Under these conditions, LSM showed reproducible but weaker downregulation of phospho-Smad1/5/8 levels in the HaCaT cells (Supplementary Figure 2B). When ALK3 was silenced and the overall phospho-Smad1/5/8 levels decreased, a weak negative effect of LSM on phospho-Smads could be recorded, whereas no effect was seen after ALK6 silencing and even a weak positive stabilization effect was seen after ALK2 silencing (Supplementary Figure 2B). The latter suggests that in the relative absence of ALK2, BMP2, which primarily signals *via* ALK3 and ALK6, generates a strong phospho-Smad1/5/8 signal, which is less sensitive to the action of LKB1. All these observations lead to the conclusion that LKB1 downregulates BMP type I receptors, especially ACVRI/ALK2 and possibly also BMPRIA/ALK3 and BMPR1B/ALK6.

If LKB1 downregulates ACVRI/ALK2, we should be able to partially or fully rescue the effects of LKB1 on BMP7 signaling by co-expressing high levels of ALK2. Indeed, in C2C12 cells, downregulation of the BRE_2_ promoter activity by LSM infection was completely rescued by concomitant co-expression of a constitutively active ALK2 at two different doses (ALK2CA, Figure 3F). Interestingly, the high level of ALK2CA expressed was downregulated only in the presence of BMP7 signaling and LSM infection (Figure 3G). Despite this weak relative decrease in receptor levels, the biological signal that reached the BRE_2_ reporter was dramatically enhanced (Figure 3F). Control experiments also showed the robust signaling capacity of the ALK2CA vector used to infect the C2C12 cells (Supplementary Figure 2C); further increase of the dose of the adenoviral vector gradually restored ALK2 signaling levels to those of cells where LKB1 was not co-expressed (Supplementary Figure 2D). These experiments suggested that BMP receptor signaling can be balanced depending on the level of its expression and/or activation.

In order to explore the mechanism whereby LKB1 affects BMP type I receptors, we investigated whether LKB1 interacted physically with the three BMP type I receptors, using co-immunoprecipitation assays. We found that LKB1 interacted with constitutively active ACVR1/ALK2 upon overexpression of both proteins (Figure 3H), and the same was true for constitutively active BMPRIA/ALK3 and BMPRIB/ALK6 (Supplementary Figure 3A). We confirmed the interaction between endogenous LKB1 and ALK2 in HaCaT cells; the interaction was constitutive and did not change upon stimulation of the cells with BMP7 for 1 h (Figure 3I). The endogenous Alk2 levels of mouse C2C12 cells are lower and did not permit successful co-immunoprecipitation analysis, which emphasizes the choice of HaCaT cells as a more robust cell model for BMP receptor signaling studies. We did not succeed in detecting the endogenous complexes between LKB1 and ALK3 or ALK6 (unpublished results).

We confirmed the ALK2-LKB1 protein association by the proximity ligation assay (PLA; [39]), which revealed complexes between adenovirally expressed constitutively active (CA) or kinase-dead (KD) human ACVR1/ALK2 with endogenous mouse Lkb1 (Figure 3J, left set of micrographs, red dots). The formation of these complexes was dramatically enhanced after adenovirus-mediated expression of LKB1 and localized predominantly to the cytoplasm (Figure 3J, right set of micrographs). Single antibody controls using anti-HA and anti-LKB1 antibodies showed minimal background signals (Supplementary Figure 3B). Unfortunately, the quality of our receptor antibodies did not allow PLA analysis of both endogenous proteins. Overall, the data show a negative role of LKB1 on BMP signaling, that involves formation of a complex between LKB1 and ACVRI/ALK2.

### LKB1 downregulates BMP type I receptors

**Figure 4:**
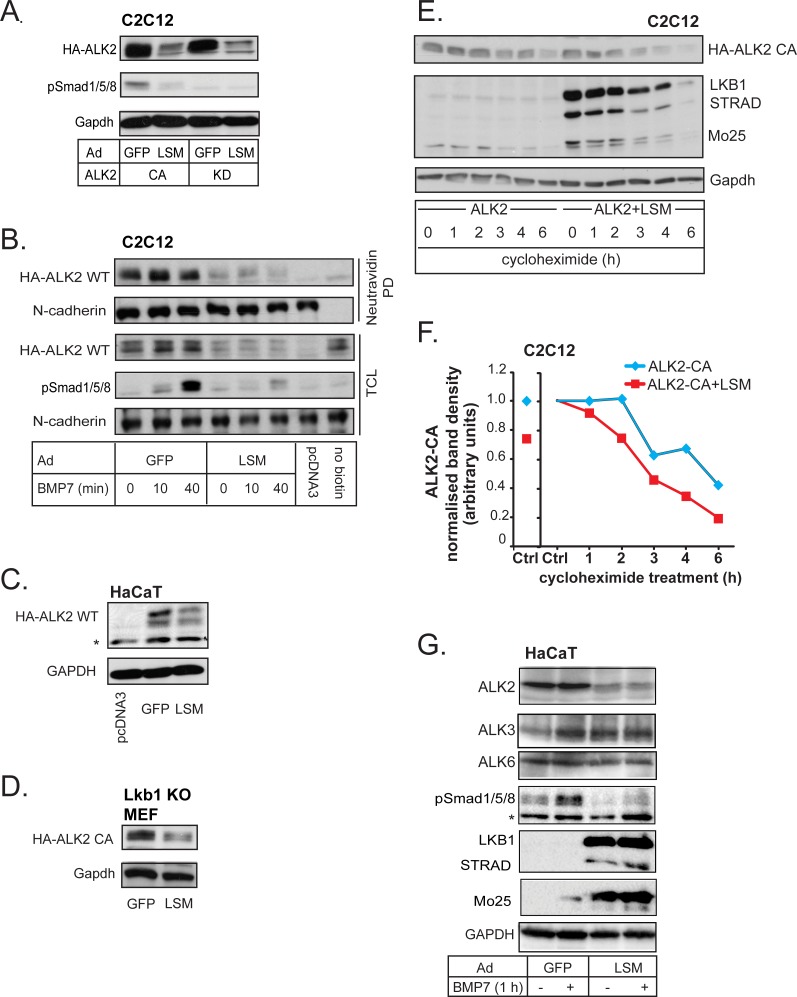
LKB1 downregulates BMP type I receptors **A.** Immunoblot of adenovirally expressed constitutively active (CA) or kinase-dead (KD) ALK2 and of endogenous phospho-Smad1/5/8 and Gapdh (loading control) in C2C12 extracts, in the presence of GFP control or LSM complex. **B.** Immunoblot measuring cell surface expression of transfected wild-type ALK2 in C2C12 cells, with adenovirally expressed GFP control or LSM complex. Cell surface proteins are detected after neutravidin pull-down (PD) of biotinylated intact cells. Phospho-Smad1/5/8 blot serves as a stimulation control for BMP7 (30 ng/ml) for the indicated time points and N-cadherin is a loading control for both biotinylated cell surface fractions (neutravidin PD) and total cell lysate (TCL). pcDNA3 empty vector and cells not treated with biotin were negative controls. **C.**, **D.** HaCaT lysates containing transfected wild-type ALK2 (C) or *Lkb1* KO MEF lysates transfected with constitutively active ALK2 (D), were immunoblotted with HA and GAPDH antibodies as loading control. Both cells have adenoviral expression of GFP or LSM complex. A star (C) indicates a non-specific protein band. **E.** Immunoblot of adenovirally expressed constitutively active (CA) ALK2 in the absence or presence of co-infected LSM proteins and endogenous Gapdh (loading control) in C2C12 extracts after treatment with vehicle (−) or 40 μg/ml cycloheximide for the indicated time periods. **F.** Graph of ALK2 protein intensities normalized to those of the corresponding Gapdh protein after densitometry of the immunoblot of panel E. The control values (0 h cycloheximide) of each experimental condition (minus or plus LSM) are shown on the left part of the diagram (left Ctrl) and are normalized to 1 on the right, main part of the diagram (right Ctrl). **G.** Immunoblots of endogenous ALK2, ALK3, ALK6, phospho-Smad1/5/8 (signaling control), GAPDH (loading control), and the three adenovirally expressed proteins of the LSM complex in HaCaT extracts after stimulation with BMP7 (30 ng/ml) for 1 h. A star shows a non-specific protein band.

To investigate the functional importance of the interaction between LKB1 and ACVR1/ALK2, we overexpressed CA or KD ACVR1/ALK2 with GFP control or LSM complex in C2C12 cells (Figure 4A). LSM expression led to a very strong suppression of the total level of constitutively active, as well as kinase-dead, ACVR1/ALK2 (Figure 4A). Cell surface expression of wild-type ACVR1/ALK2 was also greatly affected by LSM overexpression independent of BMP7 stimulation, while the level of N-Cadherin, as a control cell surface protein, was not affected (Figure 4B). Cell surface ALK2 downregulation by LSM coincided well with the loss of the phospho-Smad1/5/8 signal in time-course experiments (Figure 4B, TCL). We verified the negative effect of LKB1 on both wild-type and constitutively active ACVR1/ALK2 levels in three other cell models, i.e. HaCaT (Figure 4C), *Lkb1* knockout MEFs (Figure 4D) and HEK-293T cells (unpublished results).

We asked if LKB1 downregulates ACVR1/ALK2 expression at the mRNA level or whether it regulates the stability of the protein. LSM overexpression did not inhibit *ACVR1/ALK2* mRNA expression; in HaCaT or *Lkb1* knockout MEFs, the mRNA level of mouse *Acvr1/Alk2* or human *ACVR1/ALK2* was even weakly enhanced in the presence of LSM (Supplementary Figure 3C-E). To investigate whether LKB1 affects ACVR1/ALK2 protein stability, we used cycloheximide to block protein synthesis for up to 6 h and measured the level of constitutively active ACVR1/ALK2 over time in the presence or absence of LSM overexpression (Figure 4E, 4F). Co-expression with LSM resulted in faster turnover of ACVR1/ALK2 (Figure 4F), indicating that LSM facilitates degradation of ACVR1/ALK2. Using HaCaT cells that express all three BMP type I receptors, we could demonstrate that LSM indeed downregulated endogenous ALK2; endogenous ALK3 or ALK6 were also downregulated, but to a lower extent (Figure 4G). To more rigorously test the potential of LKB1 to downregulate BMPR1A/ALK3 and BMPR1B/ALK6, we also examined these two type I receptors after overexpression in three different cell models, C2C12, *Lkb1* knockout MEFs and HEK-293T cells (Supplementary Figure 4). HEK-293T cells were used in addition to all previous cell models due to their high efficiency of transfection; this was the only system in which we could later prove the ternary complex between LKB1, BMP receptors and inhibitory Smads. Indeed, LSM potently downregulated constitutively active and kinase dead BMPR1A/ALK3 in C2C12 cells (Supplementary Figure 4A). In transfected HEK-293T cells, LKB1 alone was able to downregulate the BMPRIB/ALK6 in a dose-dependent manner (Supplementary Figure 4B). This was confirmed in *Lkb1* knockout MEFs after LSM expression and cycloheximide-mediated block of *de novo* protein synthesis (Supplementary Figure 4C, D). Similar to the effects of LKB1 on ACVR1/ALK2 (Figure 4E, 4F), the half-life of constitutively active BMPR1B/ALK6 was significantly decreased after co-expression of LSM (notice the shift of the curve to the left in Supplementary Figure 4D). LKB1 thus seems to target all three BMP type I receptors, however, at least at the endogenous level, ALK2 seems to be more sensitive for degradation by the LKB1 signal.

### LKB1 associates with the complex between Smad7 and type I receptor and modulates receptor ubiquitinylation

**Figure 5:**
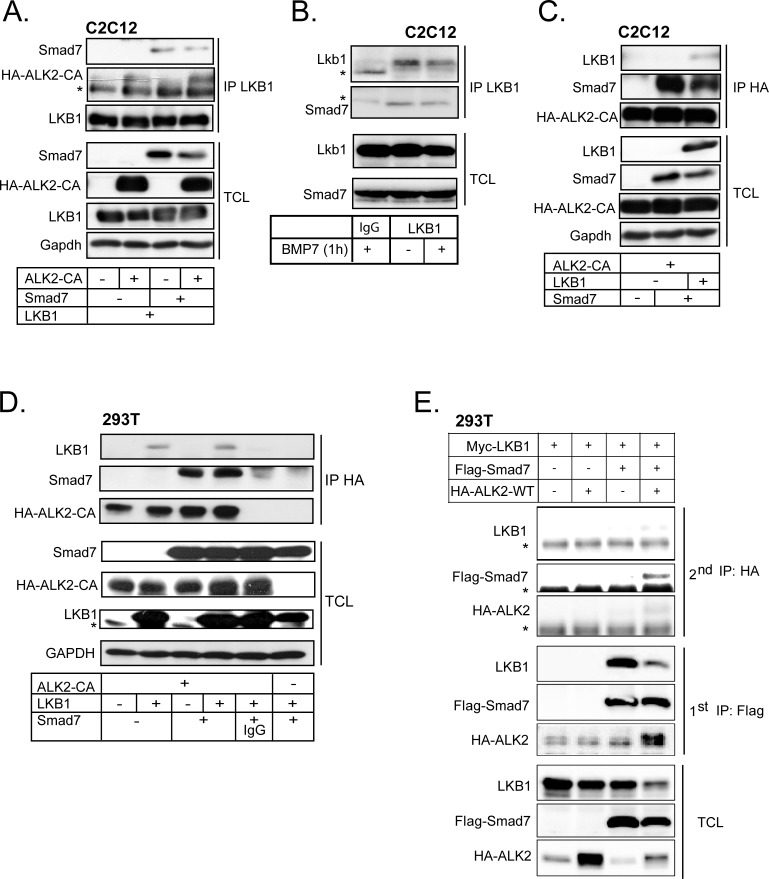
LKB1 and Smad7 associate with ACVR1/ALK2 **A.** C2C12 cells adenovirally expressing different combinations of LKB1, constitutively active ALK2 and Smad7 proteins were lysed and immunoprecipitated with anti-LKB1 antibody and immunoblotted for Smad7, HA tagged ALK2 and LKB1. Total cell lysates (TCL) were immunoblotted with the same antibodies and antibody against Gapdh, as a loading control. A star indicates a non-specific protein band. **B.** Co-immunoprecipitation assay of endogenous LKB1 with endogenous Smad7 in the absence (−) or presence (+) of BMP7 stimulation for 1 h in C2C12 cells. Extracts were immunoprecipitated with LKB1 antibody or control mouse IgG and immunoblotted for Smad7 and LKB1. Total cell lysates (TCL) were immunoblotted with the same antibodies. Stars indicate the heavy immunoglobulin protein band used for immunoprecipitation. **C.** Co-immunoprecipitation assay using C2C12 lysate and anti-HA antibody pull-down. Cells were adenovirally expressing combinations of constitutively active ALK2 receptor, LKB1 and Smad7 and immunoblotted as in panel A. **D.** HEK-293T cells were transfected with constitutively active ALK2 receptor, LKB1 and Smad7; the cell lysates were immunoprecipitated with HA antibody or control mouse IgG and immunoblotting was done as in panel A. **E.** Immunoblots of LKB1, Smad7 and wild-type ALK2 after sequential immunoprecipitation (2^nd^ IP) from cell lysates of transfected HEK-293T cells along with total cell lysate (TCL) immunoblots. The lysates after the first immunoprecipitation (1^st^ IP) are also immunoblotted. First immunoprecipitations were incubated with Flag peptide and the eluted proteins were re-precipitated with the anti-HA antibody against HA-tagged ALK2. Stars indicate non-specific protein bands.

BMP signaling induces inhibitory Smad6 and Smad7 expression as a negative feedback mechanism [40]. We therefore investigated whether Smad7 affects LKB1-induced BMP receptor stability. Smad7 has a wider effect on the BMP type I receptors, compared to Smad6 that is more specific to BMPR1A/B (ALK3/6) [41]. Using PLA, we could demonstrate an interaction between LKB1 and endogenous Smad7, in the absence or presence of constitutively active ACVR1/ALK2 (Supplementary Figure 3F, right two micrographs). PLA using single anti-LKB1 or anti-Smad7 antibody as controls showed minimal background signals (Supplementary Figure 3F, first and third micrographs). In the same cells, we then observed that transfected Smad7 co-precipitated with LKB1, with or without co-expression of constitutively active ACVR1/ALK2 (Figure 5A). ACVR1/ALK2 was co-precipitated with LKB1 together with Smad7, suggesting that they may form a ternary protein complex (Figure 5A). Endogenous Smad7 co-precipitated with endogenous Lkb1 in C2C12 cells and BMP7 stimulation for 1 h did not affect the complex (Figure 5B).

We obtained further evidence of complexes between LKB1, Smad7 and ACVR1/ALK2 by co-precipitation assay using anti-HA antibody to pull down constitutively active ACVR1/ALK2 followed by immunoblotting for Smad7 and LKB1 in both C2C12 and HEK-293T cells (Figure 5C, 5D). Smad7 co-precipitated with ALK2, and LKB1 co-precipitated with both ALK2 and Smad7 (Figure 5C). Similarly, LKB1 co-precipitated with ALK2 and with Smad7 (Figure 5D). Sequential co-precipitation first with the anti-Flag antibody against transfected Flag-Smad7, elution with Flag peptide, and then a second precipitation with anti-HA antibody against HA-tagged wild-type ALK2 demonstrated the formation of a ternary complex between the three proteins (Figure 5E). In the above co-precipitation assays, wild-type or constitutively active ALK2 receptors have been used as they both gave comparable results. Sequential immunoprecipitation was not technically possible in the C2C12 or HaCaT cells, which was the reason why we employed the highly transfectable HEK-293T cell model.

**Figure 6:**
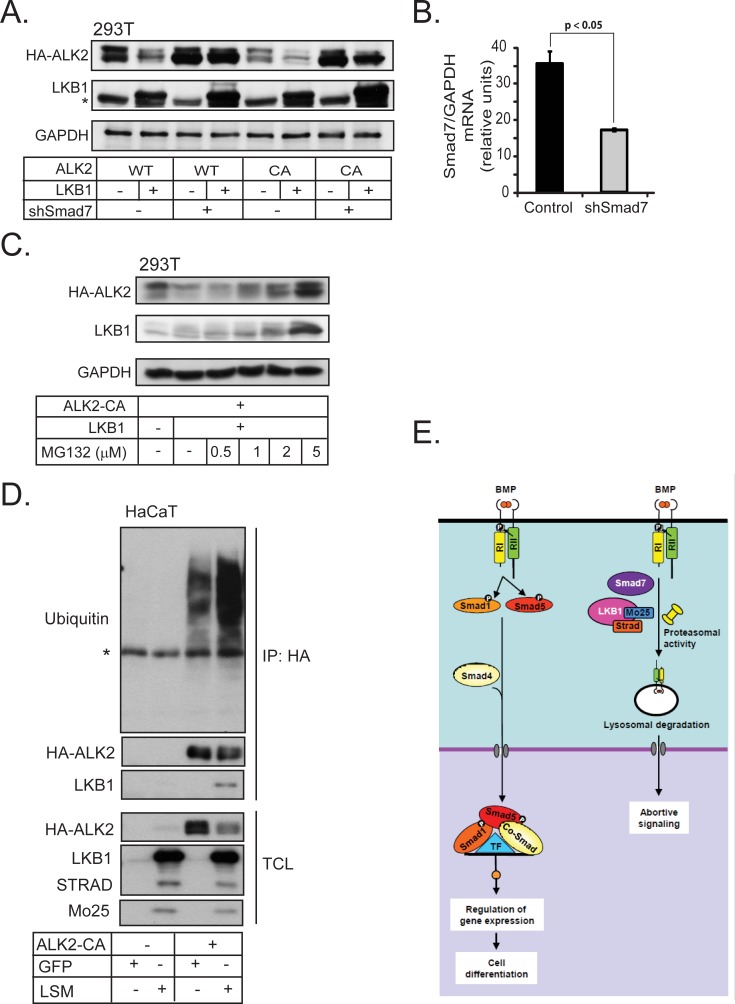
Smad7 is required for BMP type I receptor degradation by LKB1 **A.** HEK-293T cells were transfected with HA-tagged wild-type (WT) and constitutively active (CA) ALK2 together with LKB1 and short hairpin RNA targeting Smad7. Cell lysate was immunoblotted with HA and LKB1 antibodies; GAPDH was used as a loading control. A star indicates a non-specific protein band. **B.** *Smad7* mRNA expression as determined by real time PCR, as a control for Smad7 knock-down efficiency in panel A. The silenced mRNA is shown in a grey bar. Statistical significance at *p* < 0.05 between the marked measurements is indicated. **C.** Immunoblot from HEK-293T cells transfected with constitutively active (CA) ALK2 and LKB1, treated with vehicle (−) or MG132 (doses as indicated). Immunoblotting for GAPDH served as loading control. **D.** Ubiquitinylation assay of transfected HA-tagged ALK2-CA together with GFP or LSM in HaCaT cells lysates after immunoprecipitation (IP) of the transfected receptor (anti-HA antibody) followed by immunoblot for endogenous ubiquitin and transfected HA-ALK2 CA and LKB1. Total cell lysates are immunoblotted with the same antibodies. A star shows the immunoglobulin heavy chain band used for immunoprecipitation. **E.** LKB1 inhibits BMP receptor type I signaling. Normal BMP receptor signaling *via* the complex of ligand, type II receptor (RII) and type I receptor (RI) leads to Smad1 and Smad5 phosphorylation (Smad8 is omitted for simplicity) and nuclear complex accumulation together with Smad4. The nuclear Smad complex with transcription factors (TF) regulates gene expression and drives cell differentiation. The ternary complex of LKB1, STRADα and Mo25 together with Smad7 promote proteasomal and lysosomal degradation of BMP type I receptors leading to suppression of BMP signaling.

Consistent with a role for Smad7 in LKB1-mediated downregulation of ACVR1/ALK2, knock-down of Smad7 using short hairpin RNA blocked efficiently the suppressive effect of LKB1 on wild-type ACVR1/ALK2 and partially on constitutively active ACVR1/ALK2 (Figure 6A). Interestingly, LKB1 protein expression was stabilized when Smad7 was silenced (Figure 6A). The knock-down efficiency of *Smad7* on the mRNA level is shown in Figure 6B; the corresponding analysis of endogenous Smad7 protein was not successful due the quality of the antibodies available for Smad7 (unpublished results). Similar results were also obtained for BMPR1B/ALK6 after silencing the endogenous Smad7 (Supplementary Figure 4E).

We investigated whether LKB1-induced ACVR1/ALK2 degradation involved proteasomes. Treatment of HEK-293T cells with increasing doses of the proteasomal inhibitor MG132 stabilized the levels of ACVR1/ALK2 receptor in the presence of LKB1, while the level of LKB1 was also significantly enhanced (Figure 6C). In a similar manner, the impact of LKB1 on the turnover of BMPR1B/ALK6 was greatly neutralized by co-incubating the cells with the proteasomal inhibitor MG132 (Supplementary Figure 4F). In addition, ACVR1/ALK2 ubiquitinylation was strongly enhanced after co-expression of LKB1, and LKB1 associated with the ubiquitinylated receptor (Figure 6D). These results enforce a model whereby LKB1 downregulates the BMP type I receptor by recruitment of Smad7 and by enhancing poly-ubiquitinylation of the receptor, followed by degradation.

### LKB1 affects BMP signaling in the Drosophila pupal wing

**Figure 7:**
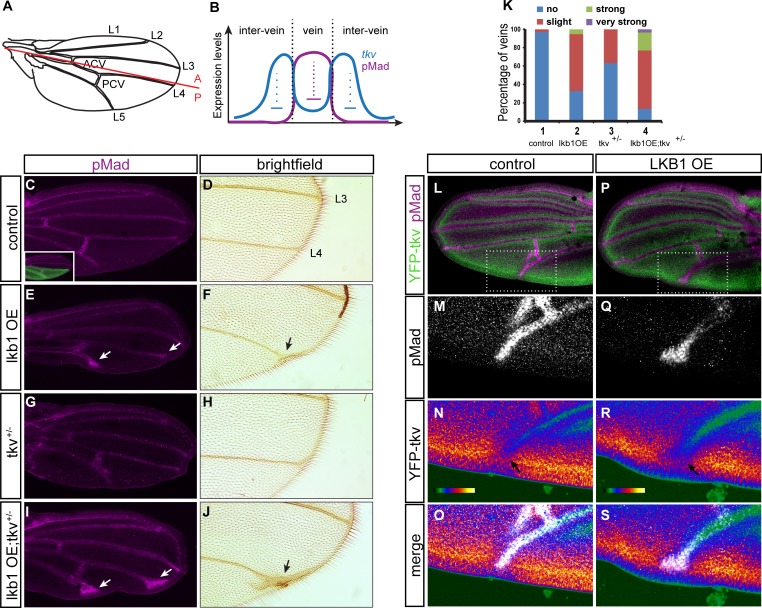
LKB1 overexpression affects BMP signaling in the *Drosophila* pupal wing **A.** Schematic of a *Drosophila melanogaster* adult wing. The five longitudinal veins (L1-L5) and the two cross-veins (ACV: anterior cross-vein; PCV: posterior cross-vein) are indicated. The border between the anterior and posterior compartment of the wing is indicated with a red line. A, anterior; P, posterior. **B.** Expression of *tkv* related to BMP activity (pMad) along the pupal vein/inter-vein region. High levels of BMP activity in cells of the future veins repress the expression of tkv. Elevated tkv levels at inter-vein cells flanking presumptive vein cells restricts BMP activity by repressing *dpp* transcription and dpp spreading. **C.**-**J.** pMad staining in *hh>Gal4>GFP* (C), *hh>Gal4>lkb1* (E), *tkv*^*strII*/+^; *hh>Gal4* (G) and *tkv*^*strII*/+^; *hh>Gal4>lkb1* (I) pupal wings and magnification of corresponding adult wings in brightfield images (D, F, H, J). The inset in panel C shows GFP staining marking the expression domain of the transgene in the posterior compartment of the wing. Arrows show longitudinal posterior vein defects. **K.** Quantification of posterior vein defects (vein 4 and 5) in *Drosophila* adult wings. 1: *hh>Gal4>GFP* (control); 2: *hh>Gal4>lkb1* (lkb1 overexpression, OE); 3: *tkv*^*strII*/+^; *hh>Gal4*; 4: *tkv*^*strII*/+^; *hh>Gal4>lkb1*. **L.**-**S.** Double immunofluorescence images for endogenous pMad and YFP-tkv in control (L-O; *hh>Gal4*) and lkb1 overexpressing (OE) pupal wings (P-S; *hh>Gal4>lkb1*). Low magnification images (L, P) with insets indicating the position of magnified areas (M-S). **O.**, **S.** The merged images show pMad pseudocolored as white for clarity. **M.**, **Q.** Isolated pMad images shown in grey scale. **N.**, **R.** The same merged images as in panels O, S, after pMad channel removal emphasizing tkv staining in intensity pseudocolor (YFP). Note the color shift from intense red-yellow to weaker red and green in panel R (arrows), indicating reduced YFP-tkv levels when lkb1 is co-expressed.

We used *Drosophila melanogaster* pupal wing development as a versatile and sensitive system for studying perturbations in BMP signaling. As explained earlier, dpp signaling specifies cell fate in pupal wings so that wing veins develop their normal pattern (Figure 7A). The type I BMP receptor tkv transduces the BMP signal and specifies the spatial limits of the signal in vein regions: tkv levels are elevated in inter-vein primordia directly abutting vein primordia and confine BMP signaling, both by repressing the expression of dpp outside vein precursors and by reducing the range of signaling by binding and sequestering dpp [42, 43] (Figure 7B). Consequently, reduction of tkv levels in hypomorphic *tkv* alleles results in a characteristic broadening of veins and in ectopic vein material adjacent to the longitudinal veins. Interestingly, we found that overexpression of lkb1 in the posterior compartment of the wing precursor (Figure 7C inset, green staining) led to similar morphological defects, such as bifurcation and thickening of the distal tips of vein 4 and 5 in adult flies (Figure 7E, 7F), when compared to control, normal wings (Figure 7C, 7D). We directly visualized the BMP activity levels in the corresponding pupal wings by immunohistochemistry (IHC) using the phospho-Smad1/5/8 antiserum that recognizes phospho-mad. In control pupal wings, BMP activity was restricted to the cells that give rise to the five longitudinal veins and the two cross-veins (Figure 7C). In contrast, overexpression of lkb1 in the posterior compartment occasionally led to the ectopic distribution of phospho-mad at the tip of the posterior vein primordia (45%, *n* = 20) (Figure 7E, arrows), suggesting that overexpression of lkb1 affects the BMP pathway and causes abnormal vein morphogenesis at the tips. Interestingly, we noted that decreasing the levels of tkv enhanced the phenotype of ectopic phospho-mad caused by overexpression of lkb1 (88%, *n* = 34) and resulted in ectopic vein morphogenesis in the adult wing (Figure 7I, 7J, 7K), a phenotype comparable to the one that has been reported in *tkv* hypomorphic alleles or after RNAi-mediated depletion of tkv [42-44]. The mutant *tkv* allele in a heterozygous setting gave no discernible phenotype as previously reported (Figure 7G, 7H; [42-44]). The cooperation between tkv loss of function and lkb1 gain of function in enhancing the ectopic vein tip morphogenesis was readily quantifiable in adult flies (Figure 7K).

To monitor effects of lkb1, we monitored the distribution and levels of YFP-tagged tkv. Co-staining for YFP-tkv and phospho-mad showed the accurate juxtaposition of the cells positive for tkv facing opposite to the cells positive for phospho-mad (Figure 7L-7O). In lkb1 overexpressing pupal wings the territory of expanded phospho-mad-positive tissue was characterized by the lack of tkv staining and a relative “repulsion” of tkv expression on tissue more distal to the longitudinal vein apex (Figure 7P-7S). In summary, these data suggest that lkb1 overexpression perturbs BMP signaling during *Drosophila* pupal wing development by limiting tkv receptor expression levels and by misregulating receptor activity as revealed by phospho-mad distribution in the pupal tissue and vein morphology in adult wings.

### LKB1 and BMP signaling profiles in human lung cancer

**Figure 8:**
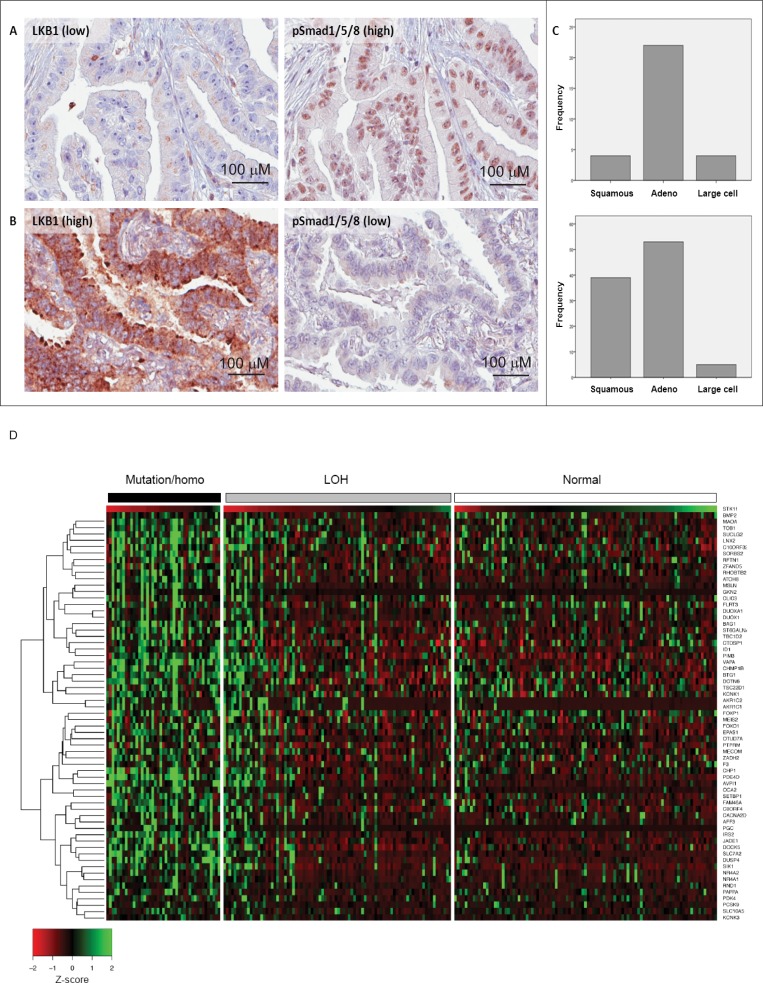
Inverse expression of LKB1 and BMP-activated Smad1 in human lung adenocarcinomas Examples of lung adenocarcinomas with low LKB1 and high phospho-Smad1/5/8 immunostaining (sample ID 164) **A.** and high LKB1 and low phospho-Smad1/5/8 immunostaining (sample ID 351) **B. C.** Frequency distribution of LKB1/phospho-Smad1/5/8 expression in non-small cell lung carcinoma histologic subgroups. Frequency refers to the number of cases: for low LKB1 and high phospho-Smad1/5/8 (*n* = 30, top), squamous *n* = 4 (13.3%), adenocarcinoma *n* = 22 (73.3%), large cell carcinoma *n* = 4 (13.3%); for high LKB1 and low phospho-Smad1/5/8 (*n* = 97, bottom), squamous *n* = 39 (40.2%), adenocarcinoma *n* = 53 (54.6%), large cell carcinoma *n* = 5 (5.2%). **D.** Heatmap of 63 genes listed in Table 1 according to Z-scores that ascribe correlation with the *LKB1* expression level. Human lung cancer samples from TCGA were classified by *LKB1* gene status: wild-type (normal), point mutation and homozygous deletion, and LOH. *LKB1* (*STK11*) gene expression is shown at the top of the gene list. Note the preponderance of green (high or no expression) in most samples with *LKB1* mutations, deletions and LOH, and the preponderance of red (low or no expression) in the samples with wild-type (normal) LKB1.

LKB1 has an established role as a tumor suppressor in the development of non-small cell lung carcinoma (NSCLC) [45]. *LKB1* gene mutations are found in 30-50% of lung cancer cell lines and 5-30% of primary human NSCLC [22, 46, 47]. On the other hand, the role of BMP in cancer is controversial. Evidence suggests that BMPs, in particular BMP2, is highly expressed in NSCLC and promotes tumor growth, invasion, and metastasis [48-51]. Based on our biochemical data, including the signaling analysis in lung adenocarcinoma A549 cells (Figure 2I and Supplementary Figure 1C), we investigated the possibility that tumor suppressive functions of LKB1 might be partially achieved through its inhibitory effect on BMP signaling, by performing immunohistochemistry on well-annotated human NSCLC surgical specimen. We analyzed total LKB1 protein levels and phospho-Smad1/5/8 as a read-out of BMP signaling in the tumor tissue, while a Smad1 antibody served as control (see Materials and Methods). High immunostaining scores for LKB1 and phospho-Smad1/5/8 were observed in 45% (*n* = 157) and 26% (*n* = 90), respectively, of the 352 NSCLCs with evaluable IHC staining. In general, an overall positive correlation was observed between the expression of LKB1 and phospho-Smad1/5/8 (*r* = 0.39; *p* < 0.01) when all lung cancer histologic subtypes were included in the analysis. However, a large number of tumors exhibited an opposite correlation, with low LKB1 and high phospho-Smad1/5/8 staining (*n* = 30) or high LKB1 and low phospho-Smad1/5/8 staining (*n* = 97) (Figure 8A, 8B). Tumors with low LKB1 and high phospho-Smad1/5/8 expression were enriched among non-squamous cancers (adenocarcinoma) compared to squamous cell carcinomas (*p* = 0.012; Figure 8C, top). A similar, but statistically less significant trend was observed in high LKB1 and low phospho-Smad1/5/8 expressing tumors (Figure 8C, bottom).

For further exploration of the mechanism, we took advantage of The Cancer Genome Atlas (TCGA) lung adenocarcinoma data and obtained a group of genes, which might be involved in the LKB1-BMP axis during lung tumorigenesis and progression (Supplementary Figure 5). First, we listed differentially expressed genes in patients with missense/truncating mutations or homozygous deletion in the *LKB1* gene compared to patients without mutation. The frequency of mutation/homozygous deletion of the *LKB1* gene was 18.7% (43/187), which confirms previous reports [22, 47]. *LKB1* mRNA expression is highly downregulated in tumors with *LKB1* mutation (*p* < 0.0001), as expected. Interestingly *BMP2* mRNA was significantly upregulated (*p* < 0.05) in the same specimen. Next, we selected those genes that exhibited a strong correlation in their expression level with *BMP2* (Spearman's correlation coefficient absolute value of 0.3 or larger; Supplementary Figure 5). We accepted as an assumption that these genes can be regulated by BMP2 in this dataset. Neither *BMP4* nor *BMP7* showed a good correlation with any known BMP target genes in this dataset (unpublished results). We therefore limited our analysis to *BMP2*, which nicely correlated with well-known BMP target genes including the *ID* family (Supplementary Table 1). We then selected the overlapping genes in the *LKB1* mutation gene list and the *BMP2* gene list (Supplementary Figure 5). Interestingly, positively correlated genes were only found among upregulated genes in the *LKB1* mutation group (63 genes in Supplementary Figure 5) and negatively correlated genes were only found among downregulated genes (66 genes in Supplementary Figure 5), which suggests that loss of *LKB1* and high expression of *BMP2* act towards the same direction in terms of gene expression. We focused on the genes that were upregulated in the *LKB1* mutation group and positively correlated with *BMP2* expression (Table 1), since these genes might be responsible for BMP2-induced tumor progression in NSCLCs with *LKB1* mutations. Although a single gene ontology term or pathway was not significantly enriched, the list contains genes related to apoptosis including anti-apoptotic function, proliferation, migration, and development (Table 1), and some of these genes are known to be involved in tumor progression of NSCLC. It was revealed that loss of heterozygosity (LOH) of the *LKB1* locus showed lower expression of *LKB1* mRNA than tumors with normal *LKB1* copy number (*p* < 0.0001), and higher expression than tumors with mutation/homozygous deletion (*p* < 0.05). The genes listed in Table 1 showed good correlation not only with *LKB1* mRNA expression but also with the group classified based on *LKB1* gene status (Figure 8D). Overall, the lung cancer analysis suggests the presence of tumor subtypes that exhibit a coupling between LKB1 and BMP signaling, which is supported by the signaling analysis in the lung adenocarcinoma cell line A549 (Figure 2I and Supplementary Figure 1C).

**Table 1:** Genes upregulated in lung cancers carrying LKB1 mutations and correlated with BMP2 expression

GO term / function	Gene symbol
Apoptosis/Cell death	*BAG1, BTG1, C8orf4, FOXO1, FOXP1, ID1, IRS2, JADE1, MECOM, PCSK9, PIM3, TSC22D1, VAPA*
Proliferation/Cell growth/ Cell cycle	*AKR1C2, AVPI1, BTG1, FOXO1, FOXP1, IRS2, JADE1, MECOM, OCA2, PDE4D, PIM3, PTPRM, SIK1, TOB1, TSC22D1, VAPA*
Development/ Differentiation	*AFF3, AKR1C1, AKR1C2, ATOH8, BTG1, CACNA2D2, CTDSP1, DUOX1, DUPXA1, EPAS1, FOXO1, FOXP1, ID1, IRS2, KCNK3, MECOM, MEIS2, NR4A2, OCA2, PAPPA, PCSK9, PCSK9, PDE4D, PGC, PTPRM, SIK1, TOB1, VAPA, ZFAND5*
Migration	*BTG1, FOXP1, ID1, IRS2, PDE4D, PTPRM, SORBS2, ZFAND5*
Adhesion	*FLRT3, MSLN, PCSK9, PTPRM, RND1, SORBS2, TBC1D2*
Angiogenesis	*BTG1, EPAS1, FOXO1, FOXP1, ID1, NR4A1, PTPRM*
Immune response/ Inflammatory response	*DCTN6, DUSP4, FOXO1, FOXP1, IRS2, RFTN1, SLC7A2*
Metabolic process/ Hormone biosynthesis	*AKR1C1, AKR1C2, DUOX1, DUOXA1, MAOA, PDK4, ST6GALNAC4, SUCLG2*
Blood coagulation	*F3, FAM46A*
Unclassified/Unknown	*C10orf32, CHMP1B, CHP1, CLIC3, DOCK5, GKN2, KCNK1, LNX2, OCA2, OTUD7A, RHOBTB2, SETBP1, SLC10A5, ZADH2*

Genes listed in Supplementary Table 1 were grouped by correlated GO terms and functions.

## DISCUSSION

This study establishes a novel mechanism of negative regulation of BMP signaling that involves the LKB1 kinase and targets the early signaling steps of receptor-mediated R-Smad phosphorylation (Figure 6E). LKB1 forms a complex with BMP type I receptors and Smad7, enhances receptor poly-ubiquitinylation and promotes proteasomal activity that can eventually lead to degradation of the receptor. Receptor downregulation leads to decreased phosphorylation of Smad1/5/8 and corresponding lowering of gene induction in response to BMP, and inhibition of BMP-induced cell differentiation (Figure 6E). No support for a role of LKB1 as a regulator of Smad stability or dephosphorylation was obtained. Since LKB1 does not affect phosphorylation of Smads by TGFβ, our observations favor a model whereby LKB1 regulates the stability of BMP type I receptors, which directly affects phosphorylation of Smad1/5/8. Additionally, this mechanism can operate during adult *Drosophila* wing morphogenesis (Figure 7), and can also be of relevance to human lung cancer (Figure 8).

LKB1 physically associates with all BMP type I receptors (Figure 5, Supplementary Figure 3A). However, we did not obtain any evidence that LKB1 directly phosphorylates the type I receptors or Smad7. In addition, attempts to link the action of LKB1 to specific members of the AMPK family, such as AMPKs or SIK, did not provide positive results. However, it remains possible that LKB1 cooperates with one of its downstream protein kinases to perform negative control of BMP signaling.

Among the three BMP type I receptors analyzed, ACVR1/ALK2 appeared to be more sensitive to the action of LKB1 at the endogenous level and in human keratinocytes, whereas BMPRIA/ALK3 and BMPR1B/ALK6 were also inhibited but to a lesser extent (Figures 3A, 4G). However, when the type I receptors were overexpressed it was readily seen that all three receptors were downregulated by LKB1. In *Drosophila*, lkb1 affected the function of the tkv receptor during wing development (Figure 7); tkv is homologous to the human BMPRIA/ALK3 and BMPRIB/ALK6 receptors, which further supports a more general negative action of LKB1 in regulating BMP receptor turnover. It is, however, possible that ALK2 has unique molecular features that make this type I receptor more sensitive to LKB1. Mutations in the *ACVR1/ALK2* gene lead to hyper-activation of ACVR1/ALK2 signaling in the genetic disease fibrodysplasia ossificans progressiva (FOP) [52] and in pediatric pontine glioma [53]. It will be interesting to investigate if activation of LKB1 may protect patients with FOP or pontine glioma. Similarly, LKB1 may have an impact on vascular diseases where defects in BMP signaling have been known to play an important role, such as pulmonary arterial hypertension caused by defects in the BMP type II receptor and hereditary hemorrhagic telangiectasia caused by defects in the type I receptor ACVRL1/ALK1 and the co-receptor endoglin [54]. So far we could not observe clear effects of LKB1 on BMP type II receptor turnover, neither did we examine the fate of ACVRL1/ALK1 as none of the cell models that we studied expressed this receptor (unpublished results).

We previously demonstrated that LKB1 can also negatively regulate the transcriptional function of Smad4, the common Smad of all pathways in the TGFβ family, including the BMP pathways [30]. Thus, during physiological BMP signaling, activation of LKB1 activity negatively regulates both BMP type I receptors and Smad4, providing an effective control mechanism.

The function of LKB1 as negative regulator of BMP signaling seems to be conserved in flies (Figure 7). The effect of lkb1 overexpression gave similar phenotype as the loss of function mutation of the dpp type I receptor tkv, and lkb1 misexpression in a domain of the wing led to localized loss of tkv in the cells apposing the wing veins. Furthermore, the observed phenotype of abnormal morphogenesis of the wing vein tips did not correlate with loss of phospho-mad signal, but rather with ectopic expression of phospho-mad (Figure 7), which is compatible with a role of lkb1 in type I receptor regulation and not directly on R-Smad regulation. This conforms to the established mechanism whereby loss of tkv activity causes a broader distribution of the dpp gradient in the responding wing tissue and correspondingly broader activation of phospho-mad, thus leading to enlarged longitudinal vein tips [18]. As presented in the introduction of this paper, lkb1 has additional functions in fly development, including regulation of body axis, epithelial polarity and lipid metabolism [23, 25-27]. Whether a crosstalk between dpp/scw/gbb signaling and lkb1 is physiologically relevant in the context of body plan, polarity or metabolic control in flies, remains to be tested in the future.

LKB1 is an established tumor suppressor and *LKB1* inactivating mutations appear to have a significant impact on sporadic non-small cell lung cancer development, since the *LKB1* gene is mutated in nearly 30% of the tumor cases examined so far [45]. In other words, the absence of LKB1 function may correlate with lung cancer development and aggressiveness. Our IHC analysis of human non-small cell lung cancers (Figure 8) confirmed that specific tumor subsets show either low LKB1 levels and high levels of BMP signaling or, inversely, high levels of LKB1 and low levels of BMP signaling. Interestingly, this inverse profile is more common in lung carcinomas of non-squamous histology. These results suggest that loss of function of LKB1 enhances BMP signaling, which may contribute to tumor progression of human lung cancer. Interestingly, this may possibly be extended beyond lung cancer and involve also metastatic breast cancer growth into the lungs of experimental animals driven by BMP7 signaling, as previously demonstrated [55]. On the other hand, it should be emphasized that findings based on tumor tissue IHC cannot provide direct cause and effect relationships. Thus, the observed levels of BMP signaling measured *via* phospho-Smad1/5/8 levels must also reflect additional regulatory events that take place during lung tumorigenesis, and which are beyond misregulation of LKB1. For example, lung tumors with very low or undetectable phospho-Smad1/5/8 levels may very well happen to suffer from BMP receptor mutations, however, the fact that at least a subset of these cancers expresses high LKB1 levels supports the model provided by our exhaustive biochemical analysis.

The possible impact of LKB1 on BMP signaling in lung cancer deserves deeper investigation. To initiate research on this front we performed meta-analysis of DNA sequencing and transcriptomic data from large cohorts of human lung adenocarcinoma deposited in TCGA (Figure 8), and confirmed previous findings on the high frequency of *LKB1* inactivation in lung adenocarcinoma, which scored at 18.7% [22, 47]. In such adenocarcinomas, a striking upregulation of *BMP2* mRNA correlated with a short gene set (63 genes) whose expression was upregulated when *LKB1* was mutated and underexpressed. We therefore propose that in human lung adenocarcinoma complete loss or underexpression of *LKB1* is linked to high BMP2 ligand production, which may then lead to the expression of factors involved in cell proliferation, apoptosis and invasiveness (Table 1). This gene set generates an interesting group of molecules worth analyzing further *via* signaling studies in human lung cancer cells. Overall, our lung cancer study suggests a clear coupling between LKB1 and BMP signaling at the mRNA and protein level that prevails in specific tumor subtypes. Minimally, the A549 lung adenocarcinoma confirms this model based on the signaling studies performed here *in vitro*.

In conclusion, this work provides new mechanistic clues about the crosstalk between LKB1 and BMP family pathways, and opens new ground for the deeper understanding of the role of these signaling proteins in tissue morphogenesis and in cancer progression.

## MATERIALS AND METHODS

### Cell culture, transfection and adenoviral infection

*Lkb1* KO mouse embryonic fibroblasts (MEF), wild-type C2C12 myoblasts, C2C12 cells stably overexpressing the BRE_2_-Luc reporter, HEK-293T cells, human lung adenocarcinoma A549 cells, and human keratinocytes (HaCaT) were cultured in Dulbecco's Modified Eagles Medium (DMEM) with 4.5 g/L glucose (Sigma Aldrich), supplemented with 10% fetal bovine serum (FBS). *Lkb1* KO MEFs [56] was a gift from R. DePinho (The University of Texas MD Anderson Cancer Center, Houston), and C2C12-BRE-luc cells [57] was a gift from P. ten Dijke (Leiden University Medical Center, Leiden). Transient transfections of cells were done using calcium phosphate [58], Lipofectamine 2000 (Invitrogen) or Fugene HD (Roche), according to standard protocols.

Transient adenoviral infections of *Lkb1* KO MEFs, C2C12, A549 and HaCaT cells were performed as previously described [59], except that cells were starved in 1% FBS/DMEM prior to adenoviral infection. Cells were infected for 18-24 h prior to stimulations with TGFβ1 (5 ng/ml) or BMP7 (30 ng/ml) in DMEM supplemented with 1% FBS. The adenoviruses for LKB1, STRADα and MO25 [60] were gifts from J. R. B. Dyck (Cardiovascular Research Centre, University of Alberta, Edmonton). AdGFP was described before [34]. Kinase-dead and constitutively active adALK2, adALK3 and adALK6 were gifts from K. Miyazono (Tokyo University Medical School, Tokyo) and were described before [31].

### Fly stocks and genetics

The UAS-LKB1 and hhGal4 fly lines [61] were provided by J. Chung (Korea Advanced Institute of Science and Technology, Daejeon) and J.E. Treisman (New York University School of Medicine, New York), respectively. All other stocks were obtained from the Bloomington *Drosophila* stock center at Indiana University (http://flystocks.bio.indiana.edu/).

Fly genotypes per figure panel were: Figure 7C: yw hsp70-flp, UAS-GFP; hh-Gal4/UAS-Gal4; Figure 7D: IF/+; hh-Gal4/UAS-Gal4; Figure 7E-7F: UAS-LKB1/IF; hh-Gal4/UAS-Gal4; Figure 7G-7H: tkvstrII/+; hh-Gal4/UAS-Gal4; Figure 7I-7J: UAS-lkb1/tkvstrII; hh-Gal4/UAS-Gal4; Figure 7L-7O: YFP-tkv/+; hh-Gal4/UAS-Gal4; Figure 7P-7S: UAS-lkb1/ YFP-tkv; hh-Gal4/UAS-Gal4.

### Plasmids

The mammalian expression vector pcDNA3 empty, and its derivatives encoding the ALK2, ALK3 and ALK6 cDNAs in wild-type, constitutively active and kinase dead forms, epitope-tagged with a haemagglutinin (HA) epitope at the C-terminus, have been described before [31, 58]. The human LKB1 plasmid was a kind gift from A. Ashworth [62]. MO25α (here abbreviated MO25) and STRADα expression vectors [29] were a gift from T.P. Mäkelä (Institute of Biotechnology, University of Helsinki, Helsinki). The LKB1 K78R catalytically inactive mutant was created by the Quickchange mutagenesis kit (Stratagene) with primers purchased from Sigma-Aldrich. The BRE-luc reporter (BRE)_2_-luc and pCMV-β-gal used for normalization of transfection efficiency have been described before [63]. The pSuper empty vector (a gift from R. Agami, Netherland Cancer Institute, Amsterdam) and the pSuper-Smad7 shRNA vector were previously described [34].

### Ligands and chemical inhibitors

Recombinant mature TGFβ1 was bought from PeproTech EC Ltd. or Biosource Inc. The TGFβ1 isoform was used throughout this study at a concentration of 5 ng/ml or lower. BMP2 was a gift of H. Lodish (Whitehead Institute for Biomedical Research, MIT, Cambridge), and BMP7 was a gift from K. Sampath (Genzyme Corp. Sanofi Co., Cambridge). The dose used for BMP7 was 30 ng/ml, unless indicated otherwise, and the doses used for BMP2 are described in the figures.

Cycloheximide (Sigma-Aldrich), an inhibitor of protein synthesis, was used at 40 μg/ml, chloroquine (Sigma-Aldrich), a lysosomal inhibitor, was used at 40 μg/ml and MG132, (Calbiochem), a proteasomal inhibitor, was used at a concentration of 50 μM unless otherwise indicated in the figures. The AMP-mimetic, 5-aminoimidazole-4-carboxamide-riboside (AICAR, Sigma-Aldrich) that activates the AMPKs, was used at a concentration of 0.1 mM. The chemical inhibitor of the mTOR kinase rapamycin (Calbiochem) was used at a concentration of 100 nM.

### Antibodies

Mouse monoclonal anti-Flag M5 antibody was from Sigma-Aldrich. Rabbit anti-phospho-Smad2 and mouse anti-Myc were home-made and have been described before [59]. Mouse anti-HA was from Roche. Rabbit anti-HA and mouse anti-LKB1 used for co-immunoprecipitation and IHC assays, mouse anti-Smad1, rabbit anti-TGFβRI/ALK5 (V-22), rabbit anti-ID1, goat anti-STRADα, goat anti-Smad7, mouse anti-β-tubulin, mouse anti-ubiquitin (P4D1), and rabbit and mouse IgGs used for control immunoprecipitations, were from Santa Cruz Biotechnology. Mouse anti-BMPRIA/ALK3, mouse anti-BMPRIB/ALK6 and goat IgG used for control immunoprecipitations and immunoblotting, were from R&D Systems, Inc. Mouse monoclonal anti-GAPDH was from Ambion. Mouse anti-E-cadherin was from Becton Dickinson Transduction Labs. Rabbit anti-phospho-Smad1/5/8, rabbit anti-phospho-AMPK (Thr172), rabbit anti-AMPKα, rabbit anti-phospho-p70 S6 kinase (Thr389), rabbit anti-p70 S6 kinase and rabbit anti-ACVRI/ALK2 were from Cell Signaling Technology, while rabbit anti-MO25 and anti-Smad1 were from Epitomics, and were used for immunoprecipitation, immunoblot and IHC assays.

### Immunoblotting, co-immunoprecipitation and ubiquitinylation assays

Proteins from transfected and/or infected and ligand-stimulated HaCaT, C2C12, HEK-293T and *Lkb1* KO MEF cells were extracted in lysis buffer (0.5% Triton X-100, 11.5 mM deoxycholic acid, 20 mM Tris-HCl pH 7.4, 150 mM NaCl, 10 mM EDTA supplemented with complete protease inhibitor cocktail from Roche) for 15 min on ice; thereafter, insoluble material was removed by centrifugation at 13,000 rpm for 15 min at 4°C. Protein concentration was determined by Bradford (BioRad) or BCA (Pierce) protein assays according to the manufacturer's protocol. Equal amounts of protein were subjected to SDS-PAGE and immunoblotting, as previously described [59]. The same protocol was used for ubiquitinylation assays after immunoblot for endogenous ubiquitin, except that the lysis buffer contained an additional 10 mM N-ethylmaleimide (Figure 6D). The reported optical density of the protein bands on the immunoblots (see Figure 4E, 4F, Supplementary Figure 4C, D) was quantified using the AIDA software (Fuji Inc.) on scanned immunoblot images from x-ray films. Specific protein band intensity (ACVR1/ALK2) was normalized to the corresponding GAPDH protein band intensity.

For co-immunoprecipitation assays with at least one transfected protein, cells were lysed in lysis buffer and incubated with rabbit anti-HA antibody, mouse anti-LKB1 antibody or rabbit IgG for 3 h, followed by incubation with protein-G magnetic beads (Invitrogen) for another 1 h at 4°C. After 5 washes with lysis buffer, the immuno-complexes were resolved by SDS-PAGE, followed by immunoblotting with antibodies, as described in the figure legends.

For endogenous co-immunoprecipitation assays protein-G magnetic beads or M280 magnetic beads (Invitrogen) were pre-coupled with goat anti-Smad7 antibody, mouse anti-LKB1 antibody, goat IgG or mouse IgG antibody in 0.5% IgG-free BSA in phosphate buffered saline (PBS) for 4 h at 4°C. Cells were lysed in lysis buffer and incubated with the pre-coupled beads overnight at 4°C and after 3 washes with lysis buffer immunocomplexes were resolved by SDS-PAGE, as described above.

For the sequential co-immunoprecipitation assay (Figure 5E), cells were lysed in lysis buffer (1% NP-40, 10 mM Tris-HCl pH 7.4, 150 mM NaCl, 1 mM EDTA, supplemented with complete protease inhibitor cocktail) and immunoprecipitated with anti-Flag M5 antibody for 2 h, followed by incubation with protein-G sepharose beads for 1 h at 4°C. Then precipitations were eluted with 100 μg Flag-peptide. Subsequently, eluates were secondary-immunopreciptated with anti-HA (Y-11) antibody overnight, followed by incubation with protein-G sepharose beads for 1 h. Immunoblotting was performed using anti-Flag, anti-HA and anti-LKB1 antibodies.

### Cell surface biotinylation assay

C2C12 or HaCaT cells were either left intact or transiently transfected with siRNA and then starved overnight in DMEM medium supplemented with 1% FBS before stimulation with 30 ng/ml BMP7 for various time periods. Cells were then washed twice in cold PBS, pH 7.3, and were incubated with sulfo-NHS-SS-biotin (0.5 mg/ml (Pierce) in PBS, pH 8.0) for 1 h at 4°C with gentle shaking. The biotinylation reaction was stopped by incubation in 50 mM Tris, pH 8.0, for 10 min on ice. After washing in PBS, pH 7.3, cells were lysed in lysis buffer and biotinylated proteins adsorbed to neutravidin agarose beads (Pierce) by 1 h incubation at 4°C. The beads were subsequently washed four times in lysis buffer, and proteins were eluted by boiling for 5 min in SDS sample buffer prior to gel electrophoresis and immunoblotting with receptor antibodies.

### Proximity ligation assay (PLA)

C2C12 cells were permeabilized with 0.2% Triton X-100 in PBS for 10 min at room temperature. Cells were washed with PBS for 2×5 min and then subjected to the PLA assay protocol of Olink Biosciences using Duolink reagents. Blocking incubation was done in DuolinkII blocking solution for 1 h at room temperature, followed by incubation in primary antibodies (1/50 dilution of rabbit anti-HA, mouse anti-LKB1 and goat anti-Smad7 antibodies from Santa Cruz Biotechnology) overnight at 4°C. Cells were washed 3 times in Buffer A. Incubation with secondary antibodies, after diluting each antibody 1:5 in DuolinkII antibody dilution buffer, at 37°C for 2 h, followed by the same washing steps. All incubations mentioned previously were done with agitation at 80 rpm.

Subsequent ligation reaction of oligonucleotides attached to each of the secondary antibodies was done using 1× Duolink ligation stock solution with Duolink ligase for 30 min at 37°C in a pre-heated humidified chamber. Cells were washed with Buffer A for 2×2 min and incubated in 1× amplification solution containing Duolink polymerase for 90 min at 37°C in a pre-heated humidity chamber. After rinsing with Buffer A, cells were stained with phalloidin 488 and Hoechst for 10 min at room temperature. Following washing with Buffer B for 2×10 min and rinsing with deionized water, cells were mounted with slowfade mounting medium (Invitrogen) and pictures taken using an epifluorescence microscope.

### siRNA transfections

C2C12 cells were treated with 20 nM siRNA oligonucleotide pools targeting mouse *Lkb1*/*Stk11* (Dharmacon ON-TARGETplus SMART pool L-044342-00-0020) or 20 nM of non-targeting control (Dharmacon ON-TARGETplus Non-targeting pool D-001810-10-20). HaCaT cells were treated with 10 nM of human si*LKB1*/*STK11* (Dharmacon ON-TARGETplus SMART pool L-005035-00-0020) or 10 nM of non-targeting control siRNA, as described above. HaCaT cells were also treated with 20 nM of human *siACVR1/ALK2* (Dharmacon ON-TARGETplus SMART pool L-004924-00-0005), 20 nM of human *siBMPRIA/ALK3* (Dharmacon ON-TARGETplus SMART pool L-004933-00-0005) and 20 nM of human si*BMPRIB/ALK6* (Dharmacon ON-TARGETplus SMART pool L-004934-00-0005). The transfection was done using SilentFect from BioRad in DMEM supplemented with 10% FBS and 48 h after transfection, cells were starved in DMEM supplemented with 1% FBS for 18-24 h prior to stimulation with BMP7.

### Real-time quantitative RT-PCR

RNA was extracted using the RNeasy kit from Qiagen. cDNA was subsequently synthesized using the iScript cDNA synthesis kit from BioRad. Real-time RT-PCR was done using iTaq SYBR green supermix with ROX from BioRad. The gene-specific primers used are as follows: mouse *Lkb1* forward 5′- GCCTCCTGAGATTGCCAATG -3′ and reverse 5′- GGTACAGGCCCGTGGTGAT-3′; mouse *Acvr1/ALK2* forward 5′-ATGGTTCTCAGACCCGACATTAAC-3′ and reverse 5′-TGCGGATGGGTTCTGATACC-3′; mouse *gapdh* forward 5′-TGTGTCCGTCGTGGATCTGA-3′ and reverse 5′-CCTGCTTCACCACCTTCTTGA-3′; mouse *Id1* forward 5′-GGACGAGCAGCAGGTAAACG-3′ and reverse 5′-TGCTCACCTTGCGGTTCTG-3′; human *ACVR1/ALK2* forward 5′- GAGGCAGCAAGCCTGGAGCA-3′ and reverse 5′- CCGCGTGCCCTCGTTCAGAG-3′; human *BMPRIA/ALK3* forward 5′- GCCAAGGGCGAAGGCCGATT-3′ and reverse 5′-TCATAAGTCCGGACCCCAGGGA-3′; human *BMPRIB/ALK6* forward 5′-CCTCCCTCTGCTGGTCCAAAGG-3′ and reverse 5′- ACCAGCTGGCTTCCTCTGTGGT-3′; and human *GAPDH* forward 5′- GGAGTCAACGGATTTGGTCGTA-3′ and reverse 5′- GGCAACAATATCCACTTTACCA.

### Luciferase assay

*Lkb1* KO MEF cells were transiently transfected with the BMP/Smad-responsive construct BRE_2_-luc for 36 h prior to stimulation with BMP7 for 18 h. pCMV-β-gal was transfected as control for normalization. C2C12 BRE-luc cells stably expressing BRE_2_-luc and β-gal were treated with 7.5 ng/ml BMP7 for luciferase assays unless differently indicated in the figures. All cells were lysed in lysis buffer containing 5 mM Tris-phosphate (Tris-HCl/KH_2_PO4) buffer pH 7.8, 2 mM dithiothreitol (DTT), 2 mM CDTA (trans-1,2-diaminocyclohexane-N,N,N′,N′ tetra-acetic acid), 5% glycerol and 1% Triton X-100. The β-Galactosidase assay was performed by mixing the cell lysate with 100 mM sodium phosphate pH 7.3, 1 mM MgCl_2_, 50 mM β-mercaptoethanol and 0.67 mg/ml of ONPG (o-Nitrophenyl β-D-Galactopyranoside) and the absorbance was monitored at 420 nm. Luciferase reporter assays were performed with the enhanced firefly luciferase assay kit from either BD PharMingen, Inc. or from Biotium Inc., according to the protocol of the manufacturers. Normalized promoter activity data are plotted in bar graphs that represent average values from triplicate determinations with standard deviations. Each independent experiment was repeated at least twice.

### Alkaline phosphatase assay

Wild-type C2C12 cells were treated with siRNA for 2 days or adenoviruses for 1 day before stimulation with 300 ng/ml of BMP7 for 3 days in DMEM containing 10% FBS. Cells were lysed in ALP lysis buffer (20 mM Tris-HCl, pH 10.5, 0.1 mM MgCl_2_, 0.01 mM ZnCl_2_, 10 mM glycine, 1% v/v Triton X-100) for 1 h on ice. Cell lysates were mixed with 6 mM p-nitrophenyl phosphate from Sigma-Aldrich at a ratio of 1 vol. substrate to 5 vol. cell lysate, and incubated at room temperature for 20 min. The resulting absorbance was measured at 405 nm. Protein concentration was measured as described above. ALP activity was determined by dividing the amount of p-nitrophenol released in nmol per min of reaction per μg of protein in the cell lysate. The graphs show average values with standard deviation bars from triplicate samples and each experiment was repeated independently at least twice.

### Drosophila wing immunocytochemistry and confocal microscopy

Pupae were dissected between 24-28 h after pupation. Pupal wings were dissected and stained according to Classen [64] and Szuperák [65], respectively. The rabbit anti-phospho-Smad1/5 (41D10; Cell Signaling Technology) antibody was used at a 1:100 dilution. YFP autofluorescence and GFP immunofluorescence in the samples were imaged with an Olympus FV1000 confocal microscope. Adult flies were collected and dissected in isopropanol. Wings were mounted in Euparal.

### Lung cancer immunocytochemistry

The *in situ* expression of LKB1 and phospho-Smad1/5/8 was assessed using immunohistochemistry on a tissue microarray constructed from formalin-fixed paraffin-embedded non-small cell lung cancer tissue, as described previously (*n* = 355) [66]. Four-micrometer sections were mounted on adhesive slides and baked for 45 min at 60°C. The slides were deparaffinized in xylene, hydrated in graded alcohols, and blocked for endogenous peroxidase in 0.3% hydrogen peroxide in 95% ethanol. For antigen retrieval, the slides were boiled in citrate buffer, pH 6.0 (PT Module Buffer, ThermoFisher Scientific, Waltham, MA USA) for 4 min at 125°C, using a pressure boiler (Decloaking chamber, Biocare Medical, Walnut Creek, CA, USA). Automated immunohistochemistry was performed using an Autostainer 480 instrument (ThermoFisher). The TMA slides were incubated with a primary antibody against LKB1 (1:100 dilution; Mouse mAb sc-32245 Santa Cruz) or phospho-Smad1/5/8 (1:100 dilution; Cell Signaling Technology) diluted in UltraAb Diluent (ThermoFisher) for 30 min at room temperature, followed by incubation with anti-rabbit/mouse UltraVision LP HRP polymer (ThermoFischer) for 30 min at room temperature. Following washing steps, the slides were developed for 10 min using diaminobenzidine and counterstained with Mayer's hematoxylin for 5 min (Histolab AB, Gothenburg, Sweden). The slides were mounted with Pertex (Histolab) and scanned using the Aperio ScanScope X (Aperio, Vista, CA, USA) to generate high-resolution digital images for evaluation of immunostainings.

Staining intensity was manually annotated on a 4-graded scale: negative (0), weak (1), moderate (2), and strong (3). The fraction of stained tumor cells was annotated as: 0-25% (1), 26-50% (2), 51-75% (3) and 76-100% (4). Duplicate tissue scores were included for each tumor on the array and an average score was calculated for each tumor with regard to intensity and fraction. For each tumor, the average scores for intensity and fraction were then multiplied, obtaining a combined score in the range 0-12, which was further dichotomized into low (0-4) and high (5-12) staining.

The Spearman correlation coefficient was calculated to assess the correlation between LKB1 and phospho-Smad1/5/8 immunohistochemical staining scores. The associations between protein expression levels (high/low) and tumor histology (squamous/non-squamous) was evaluated using the Chi-square test.

### TCGA data analysis

Gene expression data (LUAD, RNASeq2Ver2) were downloaded from The Cancer Genome Atlas data portal (https://tcga-data.nci.nih.gov/tcga/dataAccessMatrix.htm). Z-scores were calculated with average and standard deviation of all samples. Spearman's correlation coefficient, mutation, and copy number alteration data were obtained from the cBioportal site [67].

### Statistical analysis

The differences between mRNA levels or reporter luciferase activity under control, gene specific silencing and protein overexpression conditions, were evaluated statistically using a standard two-tailed *t*-test for samples with unequal variance and two-sample with equal variance, respectively. Significance is reported at *p* < 0.05.

## SUPPLEMENTARY MATERIAL FIGURES AND TABLES




