# Supplementary material for: The protein kinase LKB1 negatively regulates bone morphogenetic protein receptor signaling
Source: Oncotarget. 2015 Dec 19;7(2):1120–43. doi: 10.18632/oncotarget.6683 (PMC4811448; doi:10.18632/oncotarget.6683)
Supplement: Supplementary file 1 [file oncotarget-07-1120-s001.pdf]

The protein kinase LKB1 negatively regulates bone morphogenetic protein receptor signaling

Supplementary Information

Supplementary Table 1.

Gene lists derived from the TCGA database divided in 5 sub-tables. Heat-map generated from the data of sub-table 4 is shown in Figure 8D. See associated Excel file that includes the primary gene expression values, Pearson correlation r values and associated p values of the meta-analysis of human lung cancer transcripts obtained from TCGA.

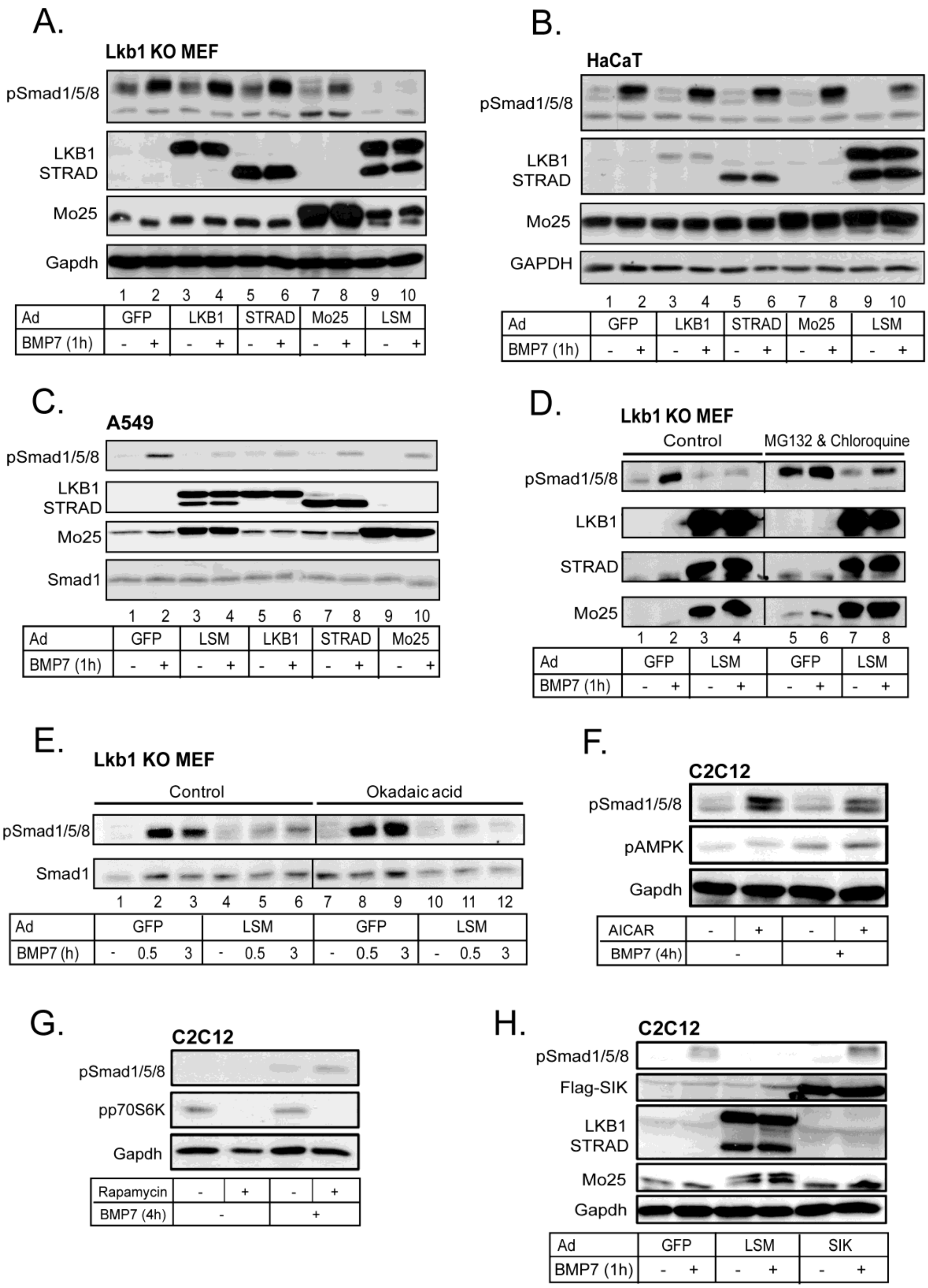

Supplementary Figure 1: LKB1 regulates Smad1/5/8 activation by BMP receptor. A-C. Immunoblots of endogenous phospho-Smad1/5/8 from *Lkb1* KO MEF **(A)**, HaCaT cells **(B)** and

A549 cells (**C**) after GFP, individual LSM component and LSM complex over-expression and followed by BMP7 stimulation (30 ng/ml) for 1 h. LKB1, STRADα and Mo25 immunoblots show the expression of endogenous and LSM infected proteins and GAPDH or Smad1 is a loading control. **D.** Immunoblot of endogenous phospho-Smad1/5/8 from *Lkb1* KO MEF pre-treated with 50 μM MG132 and 40 μg/ml chloroquine for 3 h, together with GFP or LSM complex over-expression and followed by BMP7 stimulation (30 ng/ml) for 1 h. LKB1, STRADα and Mo25 immunoblots show the expression of endogenous and LSM infected proteins. **E.** Immunoblot of endogenous phospho-Smad1/5/8 and Smad1 in *Lkb1* knockout MEF extracts after adenoviral infection and stimulation with BMP7 (30 ng/ml) for the indicated time periods in the absence or presence of treatment with vehicle (control) or okadaic acid. **F.** Immunoblot of endogenous phospho-Smad1/5/8 from C2C12 cells treated with 0.1 mM AICAR and BMP7 (30 ng/ml) for 4 h. Endogenous phospho-AMPK (Thr172) serves as control for AICAR-dependent activation and Gapdh as protein loading control. **G.** Immunoblot of endogenous phospho-Smad1/5/8 from C2C12 cells treated with BMP7 (30 ng/ml) for 4 h in the absence or presence of 100 nM rapamycin. Endogenous phospho-p70 S6 kinase (Thr389) serves as control for rapamycin efficacy and Gapdh as protein loading control. **H.** Immunoblot of endogenous phospho-Smad1/5/8 from C2C12 cells infected with GFP, LSM complex or SIK adenoviruses and followed by BMP7 stimulation (30 ng/ml) for 1 h. Flag-SIK, LKB1, STRADα and Mo25 immunoblots show the expression of endogenous and of SIK LSM infected proteins. Gapdh is the protein loading control.

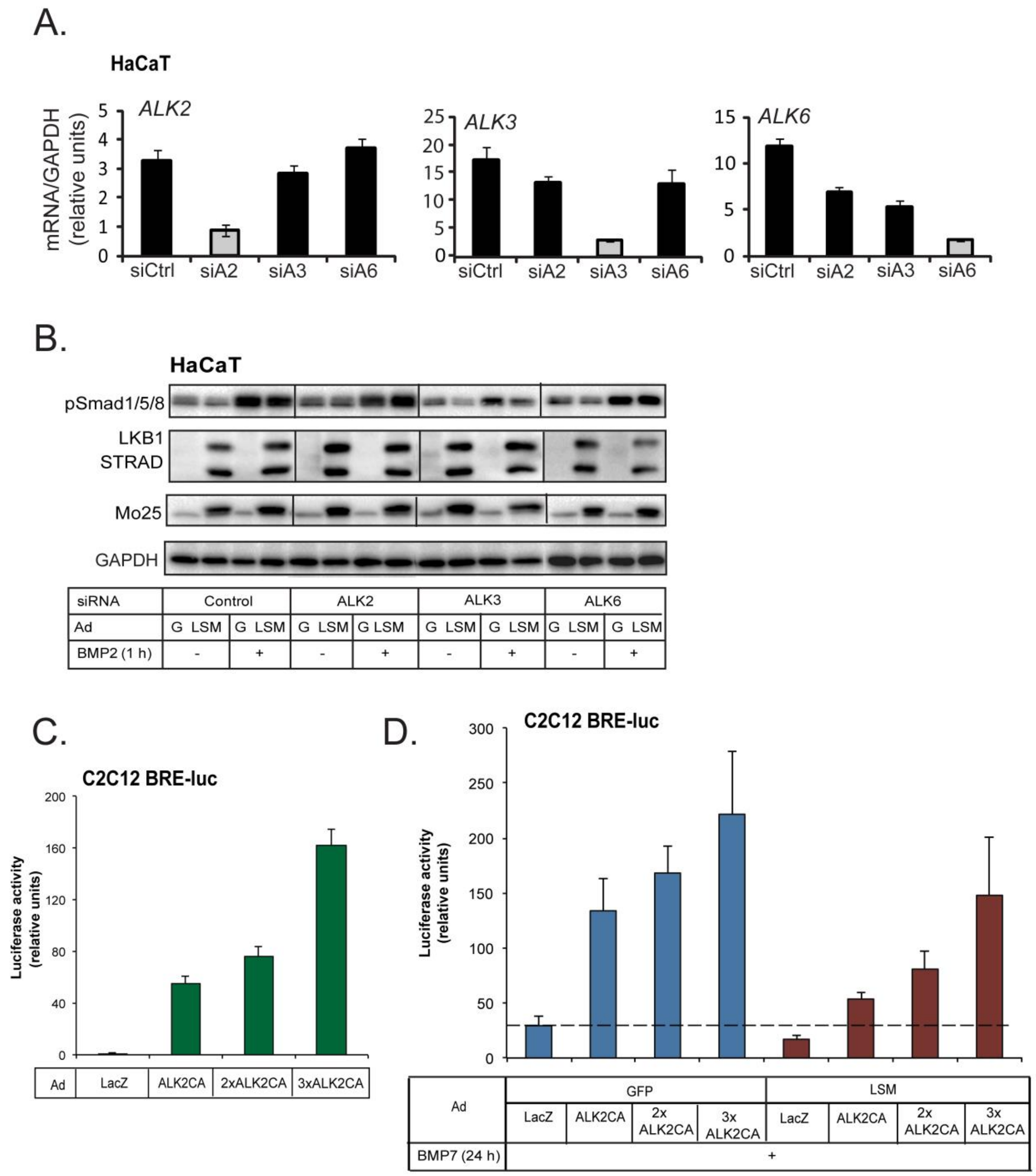

Supplementary Figure 2: LKB1 regulates BMP type I receptors in HaCaT cells. A. Real-time PCR of *ALK2*, *ALK3* and *ALK6* mRNAs served as controls for the siRNA-mediated silencing experiment used in Figure 3A. Grey bars indicate the specific mRNA that is silenced by the siRNA. Data are expressed as in Figure 1A. **B.** Immunoblot of endogenous phospho-Smad1/5/8, GAPDH (loading control) and adenovirally expressed LSM proteins in HaCaT

extracts after transfection with the indicated receptor siRNAs and stimulation with 10 ng/ml BMP2 for 1 h. **C, D.** Luciferase assays in C2C12 cells stably expressing BRE_2-luc reporter after infection with the indicated adenoviral vectors alone (**C**) or in combination with BMP7 stimulation (30 ng/ml) for 24 h (**D**)**.** The luciferase activity was normalized to the corresponding β-galactosidase activity. A dotted line in panel D. indicates the basal luciferase activity level.

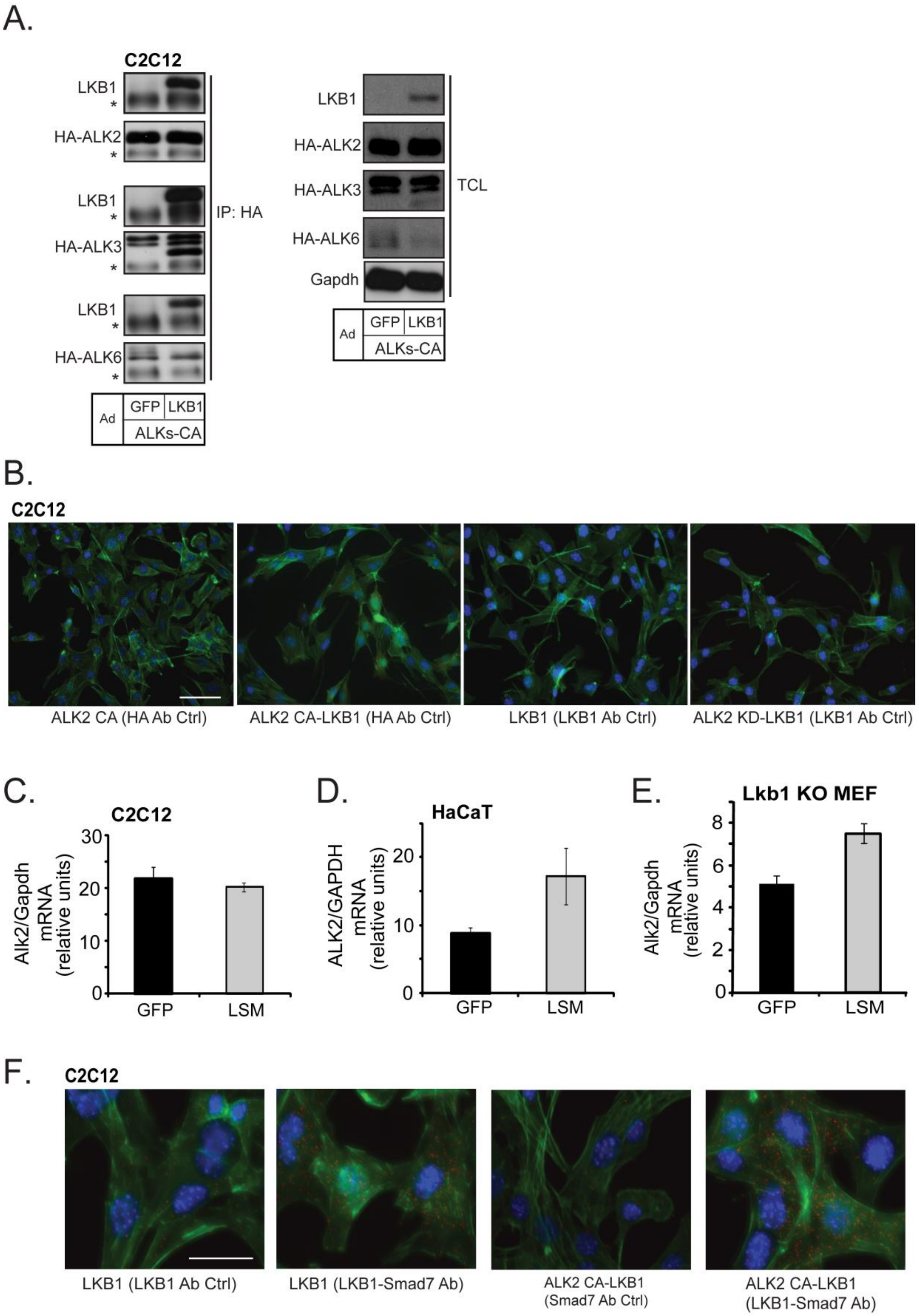
A.
C2C12
LKB1
*
HA-ALK2
*
LKB1
*
HA-ALK3
*
LKB1
*
HA-ALK6
*
IP: HA
Ad
GFP
LKB1
ALKs-CA
LKB1
HA-ALK2
HA-ALK3
HA-ALK6
Gapdh
TCL
Ad
GFP
LKB1
ALKs-CA
B.
C2C12
ALK2 CA (HA Ab Ctrl)
ALK2 CA-LKB1 (HA Ab Ctrl)
LKB1 (LKB1 Ab Ctrl)
ALK2 KD-LKB1 (LKB1 Ab Ctrl)
C.
C2C12
Alk2/Gapdh mRNA (relative units)
30
20
10
0
GFP
LSM
D.
HaCaT
ALK2/GAPDH mRNA (relative units)
20
10
0
GFP
LSM
E.
Lkb1 KO MEF
Alk2/Gapdh mRNA (relative units)
8
6
4
2
0
GFP
LSM
F.
C2C12
LKB1 (LKB1 Ab Ctrl)
LKB1 (LKB1-Smad7 Ab)
ALK2 CA-LKB1
(Smad7 Ab Ctrl)
ALK2 CA-LKB1
(LKB1-Smad7 Ab)

Supplementary Figure 3: Protein complexes between LKB1, BMP type I receptors and Smad7. A. Co-immunoprecipitation assay of adenovirally expressed constitutively active (CA) ALK2, ALK3 and ALK6 with adenovirally expressed LKB1 or negative control GFP protein. HA antibody was used to pull down ALK2, ALK3 and ALK6, and Gapdh blot served as a loading control. Stars indicate the heavy chain immunoglobulin protein bands used for immunoprecipitation. **B.** Single antibody controls (anti-HA alone or anti-LKB1 alone) for the proximity ligation assay in Figure 3J. Cells were over-expressing ALK2 receptor alone (first), LKB1 alone (third) or both ALK2 and LKB1 (second and fourth). The ALK2 receptor was constitutively active (CA) or kinase dead (KD). Green color represents the actin cytoskeleton stained with phalloidin to mark overall cell morphology and integrity, and blue color represents nuclear staining with Hoechst. A bar indicates 10 µm. **C-E.** Graphs representing the levels of *ALK2* mRNA expression with adenoviral expression of GFP (black bars) or LSM complex (grey bars) in C2C12 **(C)**, HaCaT **(D)** and *Lkb1* KO MEF **(E)** cells. mRNA expression is plotted as in Figure 1A. No statistically significant differences are measured and for this reason the graphs are not marked with a p value. **F.** In situ proximity ligation assay in C2C12 cells adenovirally expressing LKB1 with or without constitutively active (CA) ALK2, using anti-LKB1 and anti-Smad7 antibodies. The first and third micrographs represent single antibody controls, LKB1 and Smad7 antibodies respectively. The second and fourth micrographs show cells incubated with both LKB1 and Smad7 antibodies. Green color represents the actin cytoskeleton stained with phalloidin to mark overall cell morphology and integrity, and blue color represents nuclear staining with Hoechst. A bar indicates 10 µm.

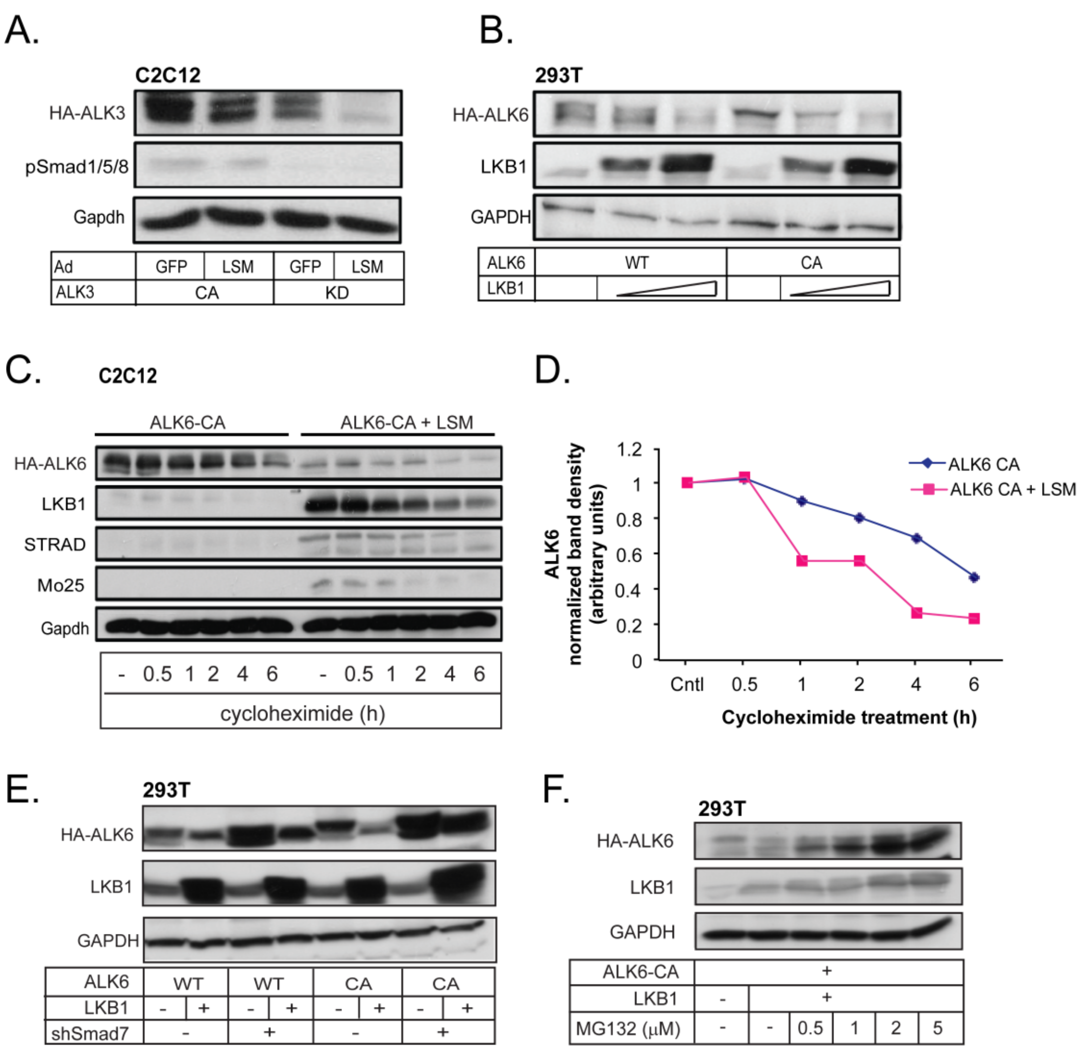

Supplementary Figure 4: LKB1 downregulates BMPR1A/ALK3 and BMPR1B/ALK6. A. Immunoblot of adenovirally expressed constitutively active (CA) or kinase-dead (KD) ALK3 and of endogenous phospho-Smad1/5/8 and Gapdh (loading control) in C2C12 extracts, in the presence of GFP control or LSM complex. **B.** Immunoblot from HEK 293T cells transfected with wild-type (WT) or constitutively active (CA) ALK6 and increasing amounts of LKB1 (triangles). Immunoblotting for GAPDH served as loading control. **C.** Immunoblot of adenovirally expressed constitutively active (CA) ALK6 in the absence or presence of co-infected LSM proteins and endogenous Gapdh (loading control) in C2C12 extracts after treatment with vehicle (-) or 40 μg/ml cycloheximide for the indicated time periods. **D.** Graph of

ALK6 protein intensities normalized to those of the corresponding Gapdh protein after densitometry of the immunoblot of panel C. The control values (0 h cycloheximide) of each experimental condition (minus or plus LSM) are normalized to 1. **E.** HEK 293T cells were transfected with HA-tagged wild-type (WT) and constitutively active (CA) ALK6 together with LKB1 and short hairpin RNA targeting Smad7. Cell lysate was immunoblotted with HA and LKB1 antibodies; GAPDH was used as a loading control. This experiment was performed under identical conditions as that shown in Figure 6A, B. **F.** Immunoblot from HEK 293T cells transfected with constitutively active (CA) ALK6 and LKB1, treated with vehicle (-) or MG132 (doses as indicated). Immunoblotting for GAPDH served as loading control.

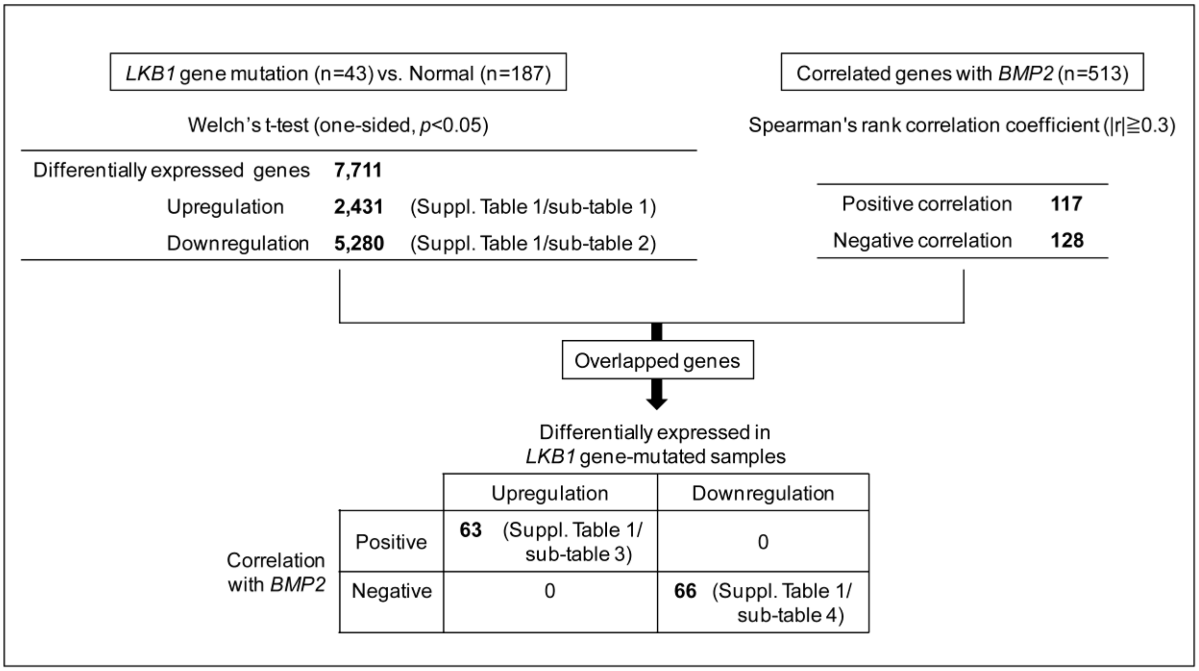

Supplementary Figure 5: Workflow of TCGA data analysis. Lung adenocarcinoma samples with *LKB1* gene mutation and/or copy number alteration were selected and compared to the *LKB1* wild-type (normal) group using the Welch's t-test to obtain differentially expressed genes (p<0.05, left column). Genes correlated with *BMP2* expression were then selected (Spearman's correlation coefficient is ≥ 0.3 and ≤ -0.3, right column). The overlapping genes in the two cohorts were finally selected (bottom table).
